# Hyperlipidemia-associated gene variations and expression patterns revealed by whole-genome and transcriptome sequencing of rabbit models

**DOI:** 10.1038/srep26942

**Published:** 2016-06-01

**Authors:** Zhen Wang, Jifeng Zhang, Hong Li, Junyi Li, Manabu Niimi, Guohui Ding, Haifeng Chen, Jie Xu, Hongjiu Zhang, Ze Xu, Yulin Dai, Tuantuan Gui, Shengdi Li, Zhi Liu, Sujuan Wu, Mushui Cao, Lu Zhou, Xingyu Lu, Junxia Wang, Jing Yang, Yunhe Fu, Dongshan Yang, Jun Song, Tianqing Zhu, Shen Li, Bo Ning, Ziyun Wang, Tomonari Koike, Masashi Shiomi, Enqi Liu, Luonan Chen, Jianglin Fan, Y. Eugene Chen, Yixue Li

**Affiliations:** 1 Key Lab of Computational Biology, CAS-MPG Partner Institute for Computational Biology, Shanghai Institutes for Biological Sciences, Chinese Academy of Sciences, Shanghai, China; 2 Center for Advanced Models for Translational Sciences and Therapeutics, University of Michigan Medical Center, Ann Arbor, MI, USA; 3 Department of Molecular Pathology, Interdisciplinary Graduate School of Medicine and Engineering, University of Yamanashi, Yamanashi, Japan; 4 Shanghai Center for Bioinformation Technology, Shanghai Industrial Technology Institute, Shanghai, China; 5 School of Life Science and Biotechnology, Shanghai Jiaotong University, Shanghai, China; 6 Department of Computational Medicine and Bioinformatics, University of Michigan, Ann Arbor, MI, USA; 7 EG Information Technology Enterprise (EGI), BasePair Biotechnology Co., Ltd., Shanghai, China; 8 Key Lab of Systems Biology, Institute of Biochemistry and Cell Biology, Shanghai Institutes for Biological Sciences, Chinese Academy of Sciences, Shanghai, China; 9 University of Chinese Academy of Sciences, Beijing, China; 10 School of Biotechnology, East China University of Science and Technology, Shanghai, China; 11 School of Life Science and Technology, Shanghai Tongji University, Shanghai, China; 12 Institute for Experimental Animals, Kobe University School of Medicine, Kobe, Japan; 13 Research Institute of Atherosclerotic Disease and Laboratory Animal Center, Xi’an Jiaotong University School of Medicine, Xi’an, China; 14 Department of Pathology, Xi’an Medical University, Xi’an, China

## Abstract

The rabbit (*Oryctolagus cuniculus*) is an important experimental animal for studying human diseases, such as hypercholesterolemia and atherosclerosis. Despite this, genetic information and RNA expression profiling of laboratory rabbits are lacking. Here, we characterized the whole-genome variants of three breeds of the most popular experimental rabbits, New Zealand White (NZW), Japanese White (JW) and Watanabe heritable hyperlipidemic (WHHL) rabbits. Although the genetic diversity of WHHL rabbits was relatively low, they accumulated a large proportion of high-frequency deleterious mutations due to the small population size. Some of the deleterious mutations were associated with the pathophysiology of WHHL rabbits in addition to the *LDLR* deficiency. Furthermore, we conducted transcriptome sequencing of different organs of both WHHL and cholesterol-rich diet (Chol)-fed NZW rabbits. We found that gene expression profiles of the two rabbit models were essentially similar in the aorta, even though they exhibited different types of hypercholesterolemia. In contrast, Chol-fed rabbits, but not WHHL rabbits, exhibited pronounced inflammatory responses and abnormal lipid metabolism in the liver. These results provide valuable insights into identifying therapeutic targets of hypercholesterolemia and atherosclerosis with rabbit models.

The European rabbit (*Oryctolagus cuniculus*) is an important experimental animal model for biomedical science. Rabbits are not only the most-used animal for the production of antibodies, but also they are widely used for studying a variety of human diseases, such as infectious disease, neoplasms, ophthalmic disease, Alzheimer’s disease, and respiratory disease^1^. Like humans, but unlike rodents, such as mice and rats, rabbits have unique features of lipid metabolism that have made them an important model for human hyperlipidemia and atherosclerosis^2^. The first experiment using rabbits to investigate atherosclerosis was performed more than a century ago^3^. When fed a diet rich in cholesterol, laboratory rabbits rapidly develop hypercholesterolemia and atherosclerosis^2^. In addition, genetic defects in low density lipoprotein receptor (*LDLR*) in Watanabe heritable hyperlipidemic (WHHL) rabbits can lead to spontaneous hypercholesterolemia and atherosclerosis, even when they are on a normal chow diet^4^,^5^. Therefore, the rabbit model has provided tremendous breakthroughs and insights into understanding the molecular and cellular mechanisms of atherosclerosis, including the discoveries of *LDLR* deficiency as a cause for human familial hypercholesterolemia^6^ and statin, the most potent lipid-lowering drug^7^, which is prescribed annually for more than 30 million hyperlipidemic patients worldwide^8^,^9^.

Despite the importance of rabbit models for the study of hypercholesterolemia and atherosclerosis, genomic and transcriptomic information related to hyperlipidemia and atherosclerosis is lacking, which hampers the use of rabbits for translational research^2^. Recently, a high-quality reference genome for the European rabbit with references to domestication and speciation was reported^10^,^11^. In the current study, we performed whole-genome sequencing on three breeds of popular experimental rabbits, wild-type New Zealand White (NZW), Japanese White (JW) and WHHL rabbits, in an attempt to identify whether there are other gene mutations or modifiers that may be involved in the pathogenesis of hypercholesterolemia and atherosclerosis in WHHL rabbits. In addition to WHHL rabbits, cholesterol-rich diet (Chol)-fed rabbits are often used as a model for the study of human hypercholesterolemia and atherosclerosis^2^. While both WHHL and Chol-fed rabbits exhibit hypercholesterolemia and atherosclerosis, the gene expression profiles of atherosclerotic lesions and livers have not been systemically investigated. Toward this goal, we conducted deep transcriptome sequencing of the aortas, livers, hearts and kidneys derived from the two hypercholesterolemic models along with wild-type control rabbits. These results provide valuable resources for the investigation of hypercholesterolemia and atherosclerosis using rabbit models.

## Results

### Whole-genome sequencing of laboratory rabbits

**Figure 1:**
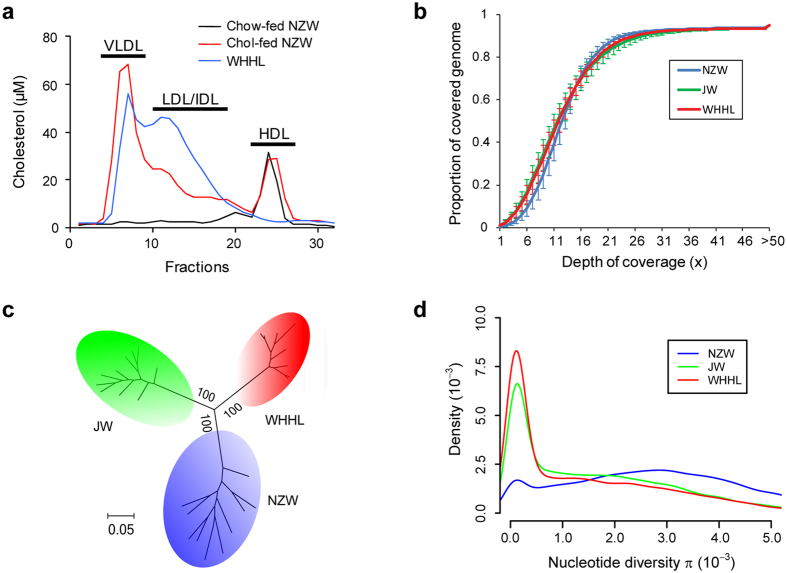
Whole-genome sequencing of laboratory rabbits. (**a**) Lipid profiles of standard chow-fed NZW, Chol-fed NZW and WHHL rabbits analyzed by high performance liquid chromatography. The Chol-fed NZW showed elevated β-VLDLs, and the WHHL rabbits showed increased LDLs and reduced HDLs. (**b**) Cumulative distribution of depth of coverage for whole-genome sequencing. The average depth of coverage was 13×  for each individual rabbit. (**c**) Phylogenic tree of the rabbits. The tree was constructed on the basis of representative SNPs with the maximum likelihood method. Bootstrap values are marked on the branch. (**d**) Distribution of nucleotide diversity *π*. The statistics were calculated for every 100 kb sliding-window across the genome.

We collected three common breeds of laboratory rabbits: NZW, JW and WHHL rabbits (Table 1). On a standard chow diet, both NZW and JW rabbits have relatively low plasma cholesterol levels compared to humans, and their cholesterol is mainly carried by high density lipoproteins (HDLs, Fig. 1a). WHHL rabbits are genetically deficient in *LDLR* function; thus, they develop hypercholesterolemia and atherosclerosis, even on a standard chow diet. Normal rabbits can also develop hypercholesterolemia and atherosclerosis when fed a diet rich in cholesterol (Chol). Although both WHHL and Chol-fed rabbits exhibit hypercholesterolemia, their lipoprotein profiles are quite different; WHHL rabbits have increased levels of LDL-cholesterol accompanied by low HDLs, while Chol-fed rabbits have increased hepatically and intestinally derived remnant lipoproteins, called β-VLDL (Fig. 1a).

We performed whole-genome sequencing of 10 rabbits for each of the three breeds (Supplementary Fig. S1), resulting in a depth of coverage of approximately 13× for each individual after alignment to the reference genome (Fig. 1b and Supplementary Fig. S2). Totally, we identified 29.8 million SNPs (Supplementary Fig. S3) and 1.6 million small indels (Supplementary Fig. S4) in the 30 genomes. Phylogenic tree building (Fig. 1c) and principal component analysis (Supplementary Fig. S5) based on genome-wide SNPs conformed distinct genetic backgrounds of the three breeds. Most of the rabbits were assumed to be unrelated except two pairs of WHHL rabbits (Supplementary Fig. S5). The genetic diversity of NZW rabbits (Table 1), measured by the nucleotide diversity *π* (Fig. 1d) and Watterson’s *θ* (Supplementary Fig. S6), was consistent with a recent report^11^. It was also higher than that of JW and WHHL rabbits, suggesting that even though all of the breeds originated from European rabbits, NZW rabbits are derived from a larger population of progenitors. Furthermore, the Tajima’s *D* (Table 1 and Supplementary Fig. S6) of NZW rabbits was positive and the largest among the three breeds, suggesting a moderate population bottleneck (sharp reduction in population size) during domestication^12^. In contrast, the Tajima’s *D* of WHHL rabbits was negative and the smallest value, which is consistent with the fact that the breed underwent a severe population bottleneck during artificial selection. The level of linkage disequilibrium was lowest in NZW rabbits and highest in WHHL rabbits (Supplementary Fig. S7), which is also in agreement with their breeding history.

### Deleterious mutations in WHHL rabbits

**Figure 2:**
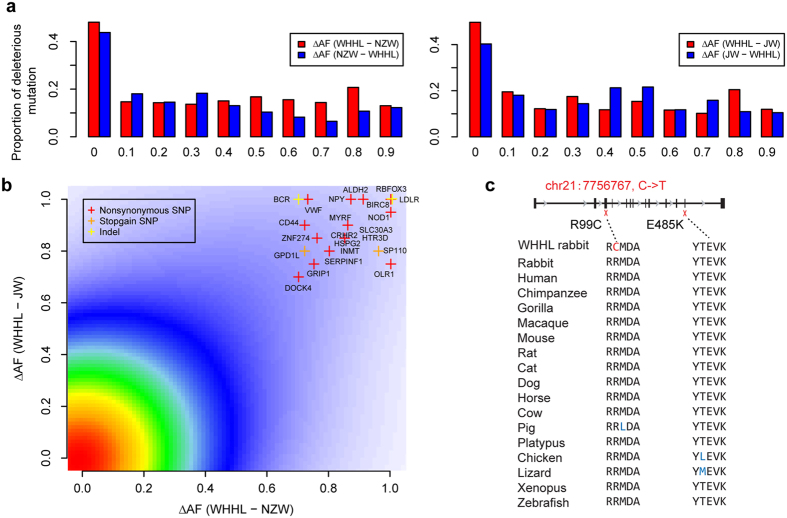
Deleterious mutations in WHHL rabbits. (**a**) Proportion of deleterious mutation by difference in allele frequency (ΔAF) between WHHL and NZW or JW rabbits. (**b**) Putative deleterious mutations in WHHL rabbits with ΔAF > 0.7 compared with both normal rabbits. Colors show the density of SNPs from high (red) to low (blue). Genes harboring deleterious mutations are highlighted. (**c**) Non-synonymous mutations in *ALDH2*. The red cross indicates the locations of mutations. R99C is a putative deleterious mutation in WHHL rabbits. E487K is a known loss-of-function mutation in humans. Both mutations occur at highly conserved sites across vertebrates.

Although WHHL rabbits are well-known for their *LDLR* mutation as a cause of hypercholesterolemia^5^, it is possible that other deleterious mutations could rise to high frequency by genetic drift due to the extremely small population size of the breed. To search for such deleterious mutations possibly involved in cardiovascular diseases, we compiled a comprehensive gene list associated with cardiovascular diseases from both the knowledge database and human genome-wide association studies (Supplementary Fig. S8). Based on the functional annotations of SNPs and indels, we predicted deleterious mutations in the prior genes using the following criteria: 1) the mutation should alter the protein sequence; 2) for non-synonymous SNPs, it should have a SIFT score <0.05^13^. We examined the proportion of deleterious mutations by the difference in allele frequency (ΔAF) between WHHL and NZW or JW rabbits (Fig. 2a). It was shown that when ΔAF > 0.4 WHHL rabbits harbor a larger proportion of deleterious mutations with high frequency than NZW rabbits, which supported our hypothesis that deleterious mutations were easier to accumulate in the WHHL breed. As the genetic diversity and hence the population size of JW rabbits were quite close to WHHL rabbits (Table 1), this result could only be observed when ΔAF > 0.8 between the two breeds.

We focused on the putative deleterious mutations specifically enriched in WHHL rabbits with a criterion of ΔAF > 0.7 compared with both normal breeds, which resulted in 24 deleterious mutations in the prior genes in addition to the known 12-bp in-frame deletion in *LDLR*^5^ (Fig. 2b and Supplementary Table S1). One of the deleterious mutations was located in *ALDH2*, the activation of which was found to decrease aortic atherosclerosis^14^. It is well-known that a loss-of-function mutation in human *ALDH2*, E487K, causes alcohol flushing and is associated with an increased risk of cardiovascular diseases^15^. Similar to this mutation in humans, the deleterious mutation that we predicted in the WHHL rabbits, R99C, occurred at a conserved site, which was invariant among all the vertebrates we examined (Fig. 2c and Supplementary Fig. S9). The mutant allele frequency remained low in the normal rabbits (NZW 10%, JW 0%) but became fixed in WHHL rabbits (100%). Another putative deleterious mutation was located in *VWF* (Fig. 2b), the mediator of blood coagulation. A mutation at the same position in the human VWF protein was reported in patients with type I von Willebrand’s disease^16^. As a hypercoagulable state was found in WHHL rabbits^17^, the role of the deleterious mutation of *VWF* deserves further investigation. There was also a deleterious mutation identified in *OLR1* (Fig. 2b), and the gene was highly expressed in the atherosclerotic lesions of WHHL rabbits^18^. These results suggested that the deleterious mutations could function as genetic modifiers in the pathophysiology of WHHL rabbits.

### Transcriptome profiling of WHHL and Chol-fed rabbits

We conducted deep transcriptome sequencing of different organs collected from both normal and hypercholesterolemic rabbits (Table 1 and Supplementary Figs S10 and 11). More than 77% of the assembled transcripts were isoforms of 15,760 known genes recorded in Ensembl (v76)^19^ (Supplementary Fig. S12). Samples originating from the same tissue were clustered together based on their gene expression profiles (Supplementary Fig. S13). We performed differential expression analysis for Chol-fed versus normal chow-fed NZW rabbits and WHHL versus JW rabbits (Supplementary Figs S14 and 15). The results showed that differentially expressed genes (DEGs) predominantly occurred in the aortas of both hypercholesterolemic models (2,719 and 1,627, respectively, false discovery rate [FDR] <0.1). In the liver, however, the transcriptional changes were notable in the Chol-fed rabbits (1,403 DEGs) but not in the WHHL rabbits (181 DEGs). The expression changes in the heart and kidney in both Chol-fed and WHHL rabbits were not remarkable compared to each wild-type rabbit.

**Figure 3:**
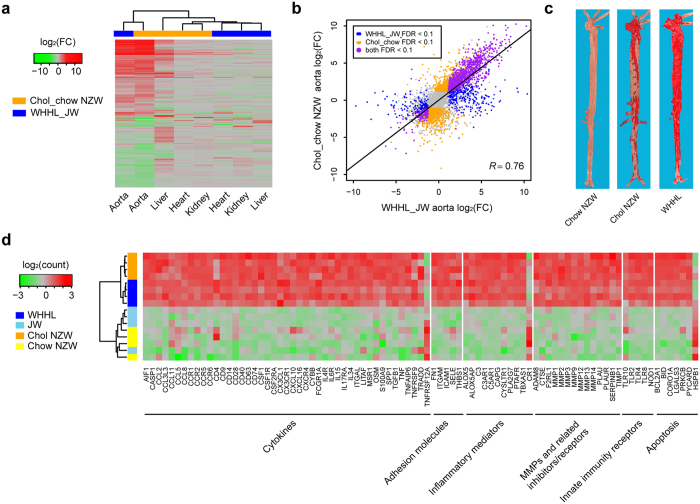
Transcriptome profiling of rabbit models with aortic atherosclerosis. (**a**) Heatmap of DEGs between Chol-fed and normal chow-fed NZW rabbits as well as between WHHL and JW rabbits. The DEGs should have FDR <0.1 in at least one comparison. Log_2_-fold changes of DEGs are illustrated by gradient colors. The transcriptional changes of Chol-fed and WHHL rabbits were similar in the aorta but distinct in the liver. (**b**) Strong positive correlation of expression changes in the aorta between Chol-fed and WHHL rabbits. The correlation coefficient was calculated for DEGs in at least one condition. (**c**) Macrographs of aortas in normal chow-fed, Chol-fed and WHHL rabbits. The aortic lesions are stained red with Sudan IV. Both Chol-fed and WHHL rabbits showed extensive atherosclerotic lesions. (**d**) Heatmap of representative DEGs responsible for inflammation responses in the aorta. The read counts were log-transformed and normalized across samples. These genes induced inflammatory responses in both Chol-fed and WHHL rabbits compared with the normal controls.

For a more detailed model-by-tissue comparison, we selected DEGs observed in at least one condition and performed clustering of the conditions based on the log_2_-fold change (logFC) of the DEGs (Fig. 3a). The result illustrates similar transcriptional responses in the aortas of Chol-fed and WHHL rabbits but different transcriptional responses in the livers of the two models. The magnitudes of aortic changes were highly positively correlated in the two models (*R* = 0.76, Fig. 3b). This result is in agreement with the pathological changes in the aortic lesions of atherosclerosis that were grossly present in both Chol-fed and WHHL rabbits (Fig. 3c). To gain functional insights into the DEGs, we performed a comparison using Ingenuity Pathway Analysis (IPA). Both function and pathway enrichment analyses supported the similarity of aortic expression changes in Chol-fed and WHHL rabbits (Supplementary Fig. S16). Notably, inflammatory responses in the lesions were significantly activated in both models (activation *z*-score = 6.28 and 6.75, respectively), which is consistent with the notion that atherosclerosis is a chronic inflammatory disease initiated by lipid deposition in the arterial wall^20^. A common set of DEGs and pathways (Supplementary Fig. S17) responsible for inflammation was observed in multiple processes of lesion development, including cytokines and chemokines along with their receptors, which are involved in monocyte adhesion, activation, and migration as well as matrix metalloproteinases (MMPs), which are responsible for extracellular matrix degradation (Fig. 3d).

### Hepatic transcriptome profiles of Chol-fed and WHHL rabbits

**Figure 4:**
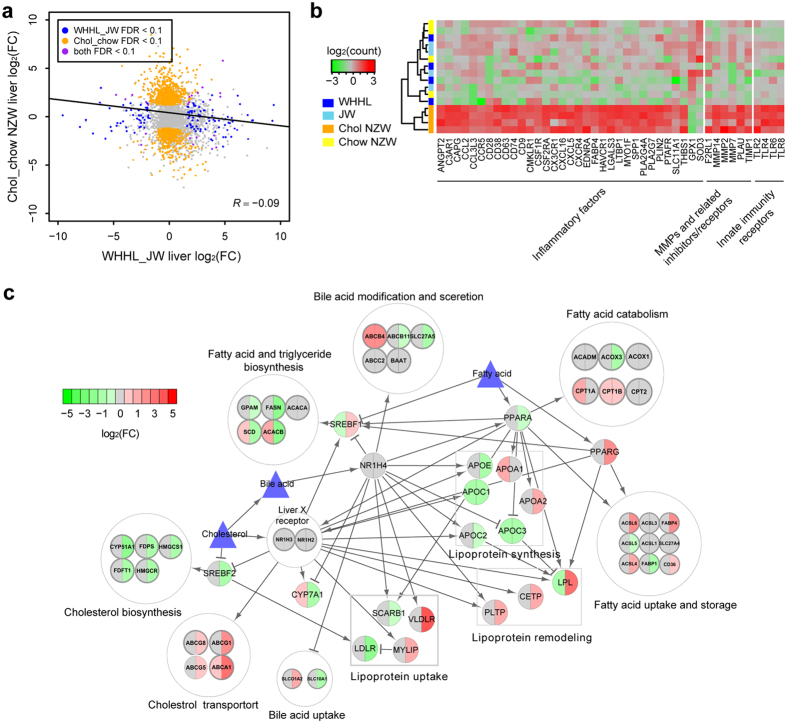
Differences in hepatic transcriptome profiles of Chol-fed and WHHL rabbits. (**a**) No positive correlation of DEGs in the liver between Chol-fed and WHHL rabbits. The correlation coefficient was calculated for DEGs for at least one condition. (**b**) Heatmap of representative DEGs responsible for inflammatory responses in the liver. The read counts were log-transformed and normalized across samples. These genes were activated in Chol-fed but not in WHHL rabbits. (**c**) Transcriptional regulatory network of lipid metabolism in the liver. The left semicircle of each gene shows the log_2_-fold change of WHHL compared with JW rabbits, while the right semicircle shows the log_2_-fold change of Chol-fed compared with normal chow-fed rabbits. Gradient colors indicate over-expression (red) or under-expression (green). Grey indicates fold change <1.5. Lines with arrowheads denote activation, and lines with crossings denote inhibition. Key transcription factors were enriched in the Chol-fed rabbits, but none were enriched in the WHHL rabbits.

Because the liver is an important organ to mediate lipid metabolism, we further investigated transcriptional changes in the livers of both Chol-fed and WHHL rabbits. As shown in Fig. 3a, both the number of DEGs and their expression patterns were dramatically changed in Chol-fed rabbits but not in WHHL rabbits. In fact, the magnitudes of the transcriptional changes in the liver displayed little correlation between the two models (*R* = −0.09, Fig. 4a). Functional analysis by IPA revealed that inflammatory responses were significantly activated in the livers of Chol-fed rabbits (activation *z*-score = 4.69). Furthermore, the DEGs mediating inflammatory responses were identified in the Chol-fed but not in the WHHL rabbits (Fig. 4b). Many inflammation-related pathways involved in aortic atherosclerosis were also invoked in the livers of Chol-fed rabbits (Supplementary Fig. S18), suggesting that cholesterol feeding can lead to hepatic injury and inflammatory changes.

To reveal the underlying metabolic impairment induced by a cholesterol-rich diet, we focused on the transcriptional control of hepatic lipid metabolism. IPA can explore upstream regulators through enrichment of their downstream DEGs, even if the regulators are not differentially expressed. This analysis identified several changes in key regulators related to cholesterol and fatty acid homeostasis in the livers of Chol-fed rabbits, including liver X receptors (*NR1H2* and *NR1H3*), farnesoid X receptor (*NR1H4*), *SREBF1*, *SREBF2*, *PPARA* and *PPARG*^21^ (Fig. 4c and Supplementary Fig. S18). Liver X receptors are cholesterol-activated nuclear receptors, which target genes involved in reverse transport of excess cholesterol from peripheral tissues to the liver^22^. *ABCA1* (FDR = 5.87 × 10^−8^), *ABCG1* (FDR = 9.74 × 10^−5^), *PLTP* (FDR = 0.05) and *LPL* (FDR = 3.89 × 10^−12^), the target genes responsible for cholesterol transport and lipoprotein remodeling, were significantly up-regulated in the livers of Chol-fed rabbits (Supplementary Fig. S19). Another target gene, *MYLIP,* (FDR = 0.02) was also significantly overexpressed, which could trigger the ubiquitylation and degradation of LDLR^22^. *SREBF2* is a transcriptional factor responsible for cholesterol synthesis and is activated by low cellular cholesterol levels^23^. Not surprisingly, both *SREBF2* (FDR = 0.05) and its target genes, *FDFT1* (FDR = 0.02) and *CYP51A1* (FDR = 0.02), were significantly repressed in the livers of Chol-fed rabbits (Supplementary Fig. S20). The expression of *PPARG*, a fatty acid-activated nuclear receptor^24^, was significantly induced (FDR = 9.37 × 10^−4^) along with its target genes, such as *FABP4* (FDR = 0.04) and *ACSL4* (FDR = 0.09), suggesting the occurrence of active fatty-acid uptake and storage (Supplementary Fig. S21). The regulation of other transcription factors was more complex, probably due to the reciprocal interactions among factors and metabolites^21^. In contrast to the Chol-fed rabbits, none of these genes was changed in the livers of WHHL rabbits, suggesting that dietary cholesterol plays an important role in inducing these changes.

## Discussion

In the current study, we performed whole-genome sequencing with DNA from three breeds of popular laboratory rabbits (NZW, JW and WHHL rabbits) and catalogued millions of variants for these breeds. We also performed deep mRNA sequencing of the aorta, liver, heart and kidney from the rabbits and provided the most comprehensive transcriptome assembly to date. The completion of rabbit genome and transcriptome information will facilitate many facets of rabbit studies to investigate human diseases and also drive the generation of transgenic and knock-out rabbits^2^.

The whole-genome sequences of the three experimental rabbit breeds were consistent with their breeding history. Particularly, the severe bottleneck during artificial selection of WHHL rabbits gave rise to many deleterious mutations with high frequency in the breed. In addition to the *LDLR* functional deficiency, some of the deleterious mutations could also play a role in the pathogenesis of hypercholesterolemia and atherosclerosis in WHHL rabbits. For example, a high-frequency deleterious mutation was predicted at a quite conserved site on *ALDH2*, which was previously reported to play a cardio-protective role via both the elimination of toxic aldehydes under oxidative stress and the bioactivation of nitroglycerin to nitric oxide^25^. Recent studies found that Alda-1, an activator of *ALDH2*, can reduce myocardial infarct size induced by acute ischemia^26^. Administration of Alda-1 decreases aortic atherosclerosis in *Apoe* knockout mice^14^. Because WHHL rabbits suffer from hypercholesterolemia, atherosclerosis, myocardial infarction^27^, insulin resistance^28^ and visceral adipose accumulation^29^, further studies are required to elucidate the pathophysiological significance of the deleterious mutations.

Although rabbits are frequently used to study human atherosclerosis, gene expression profiling of aortic and liver lesions has not been systemically elucidated. We conducted transcriptome sequencing of these two tissues of WHHL and Chol-fed NZW rabbits and compared them with those of control rabbits. Quantification of the transcriptome allowed us to identify more than 2,000 DEGs in aortic atherosclerotic lesions of WHHL and Chol-fed rabbits, as well as more than 1,000 DEGs in the livers of Chol-fed rabbits. We found that many inflammation-related genes were upregulated in aortic atherosclerotic lesions of both WHHL and Chol-fed rabbits, which is consistent with the notion that atherosclerosis is essentially evoked by inflammation^30^. In this regard, many cytokines and chemokines, along with their receptors and apoptosis signaling pathways, were markedly upregulated. In addition, many MMPs were upregulated in the lesions, suggesting that they are involved in lesion formation^31^. Nevertheless, gene expression profiles of the lesions were similar between WHHL and Chol-fed rabbits, suggesting that regardless of the different types of hypercholesterolemia in WHHL (predominated by LDLs) and Chol-fed (predominated by β-VLDL or remnant lipoproteins) rabbits, the process of lesion development occurs in a similar pathway, namely, inflammation initiated by lipid deposition in the intima. However, it should be noted that Chol-fed rabbits, but not WHHL rabbits, also exhibited a pronounced inflammatory response and abnormal lipid metabolism in the liver, suggesting that exogenous dietary cholesterol feeding not only elevates the plasma levels of cholesterol but also induces hepatic dysfunction. In fact, it has been reported that feeding rabbits with 1% cholesterol for 8 weeks can induce non-alcoholic fatty liver disease^32^.

In conclusion, we have successfully characterized the whole-genome variants and transcriptome profiles of the three breeds of laboratory rabbits. In a future study, we will investigate whether these deleterious mutations and DEGs play any roles in the pathogenesis of various disorders in the hyperlipidemic rabbits. These resources will be vital in identifying therapeutic targets for the treatment of hyperlipidemia and atherosclerosis.

## Methods

### Sample collection

NZW rabbits were purchased from Covance Com., USA, and JW rabbits were purchased from Japan SLC Inc. (Shizuoka, Japan) and WHHL rabbits were provided by Kobe University School of Medicine, Japan. The rabbits were fed a normal chow diet. Animals were euthanized by overdose injection of pentobarbital solution. We collected liver tissue for extracting DNA for genome sequencing (*n* = 10). For the transcriptome profiling, we also used NZW rabbits fed with a cholesterol-rich diet containing 0.3% cholesterol and 3% soybean oil for 16 weeks, which is commonly used for studying atherosclerosis. The liver, kidney, heart and aortic arch were collected for extracting total RNA for transcriptomic analysis (*n* = 4). All animal experiments were performed with the approval of the Animal Care and Use Committee of the University of Yamanashi, Kobe University and University of Michigan. The methods were carried out in accordance with relevant and approved guidelines and regulations.

### Genome and RNA Sequencing

Genomic DNA was extracted from 200 μl liver tissue lysate with the QIAamp Blood DNA mini kit (Qiagen, Germany) according to the manufacturer’s manual. DNA quality and integrity were controlled by the A260/280 ratio, agarose gel electrophoresis using Qubit 2.0 (Life Technologies, USA) and a Bioanalyzer 2100 (Agilent, Germany). For sequencing the library preparation, 1 μg of genomic DNA was sheared to fragments of 300–400 bp, end-repaired, A-tailed and ligated to Illumina sequencing adapters. The ligated products were selected for a length of 400–500 bp on a 2% agarose gel and amplified by LM-PCR. The library was sequenced on an Illumina HiSeq2000 with 2 × 100 bp paired-end mode, which was controlled by HiSeq Control Software. Total RNA sample quality and integrity were controlled with a Bioanalyzer 2100 (Agilent, Germany). For the library preparation, 3 μg total RNA was captured by Dynabeads Oligo (Life Technologies, USA), sheared to fragments of 200 bp, and reverse transcribed by the SuperScriptIII cDNA Synthesis Kit (Life Technologies, USA). cDNA was end-repaired, A-tailed and ligated to Illumina sequencing adapters and amplified by PCR. Library preparation was performed with the TruSeq RNA LT V2 Sample Prep Kit (Illumina, San Diego). The sequencing library was qualified by Qubit 2.0 (Life Technologies, USA) and a Bioanalyzer 2100 (Agilent, Germany) and then sequenced on an Illumina HiSeq2000 with 2 × 100 bp paired-end mode, which was controlled by HiSeq Control Software.

### DNA variant calling

Raw sequencing reads were processed by in-house scripts to filter low quality reads and trim low quality bases (quality <20) at the 3′ end of reads. Remaining reads were aligned to the rabbit reference genome, oryCun2 by BWA-MEM (v0.7.4)^33^, with default parameters. Aligned reads were sorted and summarized by SAMtools (v0.1.9)^34^, where duplicated reads were removed. SNPs and genotypes were called by the SAMtools pipeline^34^ with recommended parameters. The rabbits belonging to the same breed were combined for variant calling. SNPs with a depth of coverage lower than 20 or higher than 300 were filtered. Small indels were called by Dindel (v1.0.1)^35^, which performed local realignment around candidate indels. The recommended workflow for diploid samples was adopted. Only indels occurring in at least two samples were selected for realignment, and the maximum number of reads in a realignment window was 50. Variants of different rabbits were merged by VCFtools (v0.1.11)^36^. Functional effects of the variants after filtering were annotated by ANNOVAR (v2013-06-21)^37^, using Ensembl (v76)^19^ genes as the database. Predictions of non-synonymous SNPs on protein function were downloaded from the SIFT database for the rabbit genome (http://sift-db.bii.a-star.edu.sg/, v2014-10-07)^13^.

### Population genetics analysis

Genetic relationships between all pairwise rabbits were estimated by KING^38^, which reported the proportion of SNPs with zero identical-by-state and calculated kinship coefficients. PCA clustering was performed by GCTA (v1.13)^39^. The phylogenetic tree was constructed by SNPhylo (v2014-07-01)^40^, which selected representative SNPs based on LD blocks and produced maximum likelihood trees. Standard population genetic statistics, including nucleotide diversity *π*, Watterson’s *θ* and Tajima’s *D* were calculated using packages in Bioperl (v1.6.1)^41^ and PopGenome (v2.0.8)^42^. Consecutive sliding windows with fixed sizes along the genome were adopted for the calculation. Correlation coefficients between two SNPs were calculated by HaploView (v4.2)^43^. A random set of 500,000 SNPs from each breed was used for genome-wide LD estimation.

### Prior genes and ortholog mapping

Prior genes involved in cardiovascular diseases were retrieved from QIAGEN’s Ingenuity Pathway Knowledge Base (accessed 2014-09-28), which is a data repository of manually reviewed biological interactions and functional annotations. In addition, cardiovascular disease-associated genes provided by the NHGRI GWAS Catalog^44^ were also incorporated. The human genes were mapped to matched rabbit genes using orthologous information from TreeFam (v9)^45^ and Ensembl (v76)^19^.

### RNA expression quantification

Quality of RNA sequencing reads were checked by the NGS QC Toolkit (v2.3.2)^46^. Reads were filtered if the quality score of more than 75% bases was below 20. Bases from the 3′ end were trimmed if their quality was below 20, and reads shorter than 40 bases were discarded after trimming. High quality reads were mapped to the rabbit reference genome (OryCun2) by TopHat2 (v2.0.8)^47^, using the Ensembl transcript annotations (v76)^19^ as the reference. Transcripts were assembled and merged by Cufflinks (v2.0.2)^48^ with the guidance of the reference. Seven samples with extremely large gene numbers (>50,000) were ignored in the assembly merging. Assembled genes and transcripts with extremely low expression level (FPKM <0.5) in all samples were removed. Reads that mapped to genes were counted by HTSeq (v0.6.0)^49^. Raw counts were normalized by DESeq (v1.16.0)^50^, and DEGs based on the negative binomial distribution were detected with this package (FDR <0.1). The magnitude of expression changes was measured by the log_2_-fold change of normalized counts. Hierarchical clustering was performed with the heatmap.2 function in R (http://www.r-project.org/).

### Functional analysis of DEGs

Function, pathway and regulator enrichment of DEGs were analyzed through the use of QIAGEN’s Ingenuity Pathway Analysis (IPA, http://www.ingenuity.com/products/ipa, accessed 2014-11-26). Human annotations of the rabbit orthologs were used for analysis of the data sets. IPA’s default cutoffs were adopted for activation state prediction (activated: activation *z*-score >2; inhibited: activation *z*-score <−2; overrepresented: *P*-value < 0.05). The regulation network of lipid metabolism in the liver was visualized by Cytoscape (v3.2.0)^51^ and its plugin enhancedGraphics (v.1.0.1)^52^.

## Additional Information

Accession codes: All sequencing data from this study have been submitted to the NCBI Sequence Read Archive (SRA; http://www.ncbi.nlm.nih.gov/sra) under accession number SRP053211 (genome sequencing) and SRP053164 (transcriptome sequencing). 

**How to cite this article**: Wang, Z. *et al.* Hyperlipidemia-associated gene variations and expression patterns revealed by whole-genome and transcriptome sequencing of rabbit models. *Sci. Rep.* **6**, 26942; doi: 10.1038/srep26942 (2016).

## Supplementary Material

Supplementary Information

## Figures and Tables

**Table 1:** Experimental design and sequencing data.

Breed	DNA					RNA			
	Sample size	Depth of coverage per sample	Nucleotide diversity *π*	Watterson *θ*	Tajima *D*	Diet	Tissue collected	Sample size	Data (Gb) per tissue per sample
NZW	*n* = 10	13.34	2.80 × 10^−3^	2.26 × 10^−3^	0.77	Standard chow diet	Aorta, liver, heart, kidney	*n* = 4	5.10
						0.3% cholesterol diet	Aorta, liver, heart, kidney	*n* = 4	5.13
JW	*n* = 10	12.94	1.61 × 10^−3^	1.37 × 10^−3^	−0.01	Standard chow diet	Aorta, liver, heart, kidney	*n* = 4	5.93
WHHL	*n* = 10	12.75	1.44 × 10^−3^	1.19 × 10^−3^	−0.08	Standard chow diet	Aorta, liver, heart, kidney	*n* = 4	5.70
