# Supplementary material for: Hyperlipidemia-associated gene variations and expression patterns revealed by whole-genome and transcriptome sequencing of rabbit models
Source: Sci Rep. 2016 Jun 1;6:26942. doi: 10.1038/srep26942 (PMC4887883; doi:10.1038/srep26942)
Supplement: Supplementary Information [file srep26942-s1.pdf]

Supplementary information

Hyperlipidemia-associated variations and expression patterns revealed by whole-genome and transcriptome sequencing of rabbit models

Zhen Wang [1#], Jifeng Zhang [2#], Hong Li [1#], Junyi Li [1#], Manabu Niimi [3], Guohui Ding [1,4], Haifeng Chen [4,5], Jie Xu [2], Hongjiu Zhang [6], Ze Xu [7], Yulin Dai [8,9], Tuantuan Gui [8,9], Shengdi Li [8,9], Zhi Liu [8,9], Sujuan Wu [4,10], Mushui Cao [4,11], Lu Zhou [8,9], Xingyu Lu [5], Junxia Wang [5], Jing Yang [4,10], Yunhe Fu [8,9], Dongshan Yang [2], Jun Song [2], Tianqing Zhu [2], Shen Li [3], Bo Ning [3], Ziyun Wang [3], Tomonari Koike [12], Masashi Shiomi [12], Enqi Liu [13,14], Luonan Chen [8], Jianglin Fan [3,14*], Y. Eugene Chen [2*], Yixue Li [1,4,5,10,11*]

1. Key Lab of Computational Biology, CAS-MPG Partner Institute for Computational Biology, Shanghai Institutes for Biological Sciences, Chinese Academy of Sciences, Shanghai, China
2. Center for Advanced Models for Translational Sciences and Therapeutics, University of Michigan Medical Center, Ann Arbor, MI, USA
3. Department of Molecular Pathology, Interdisciplinary Graduate School of Medicine and Engineering, University of Yamanashi, Yamanashi, Japan
4. Shanghai Center for Bioinformation Technology, Shanghai Industrial Technology Institute, Shanghai, China
5. School of Life Science and Biotechnology, Shanghai Jiaotong University, Shanghai, China
6. Department of Computational Medicine and Bioinformatics, University of Michigan, Ann Arbor, MI, USA
7. EG Information Technology Enterprise (EGI), BasePair Biotechnology Co., Ltd., Shanghai, China

8. Key Lab of Systems Biology, Institute of Biochemistry and Cell Biology, Shanghai Institutes for Biological Sciences, Chinese Academy of Sciences, Shanghai, China
9. University of Chinese Academy of Sciences, Beijing, China
10. School of Biotechnology, East China University of Science and Technology, Shanghai, China
11. School of Life Science and Technology, Shanghai Tongji University, Shanghai, China
12. Institute for Experimental Animals, Kobe University School of Medicine, Kobe, Japan
13. Research Institute of Atherosclerotic Disease and Laboratory Animal Center, Xi'an Jiaotong University School of Medicine, Xi'an, China
14. Department of Pathology, Xi'an Medical University, Xi'an, China

[#] These authors contributed equally to this work.

[*] To whom correspondence should be addressed.

E-mail: jianglin@yamanashi.ac.jp (J. Fan), echenum@umich.edu (Y.E. Chen), yxli@sibs.ac.cn (Y. Li)

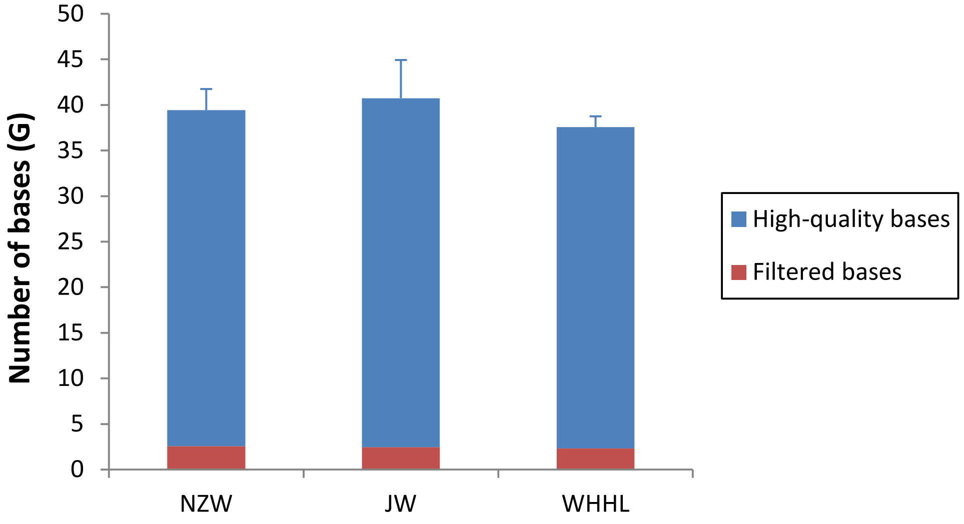

Supplementary Figure S1. Summary of whole-genome sequencing data and quality control.

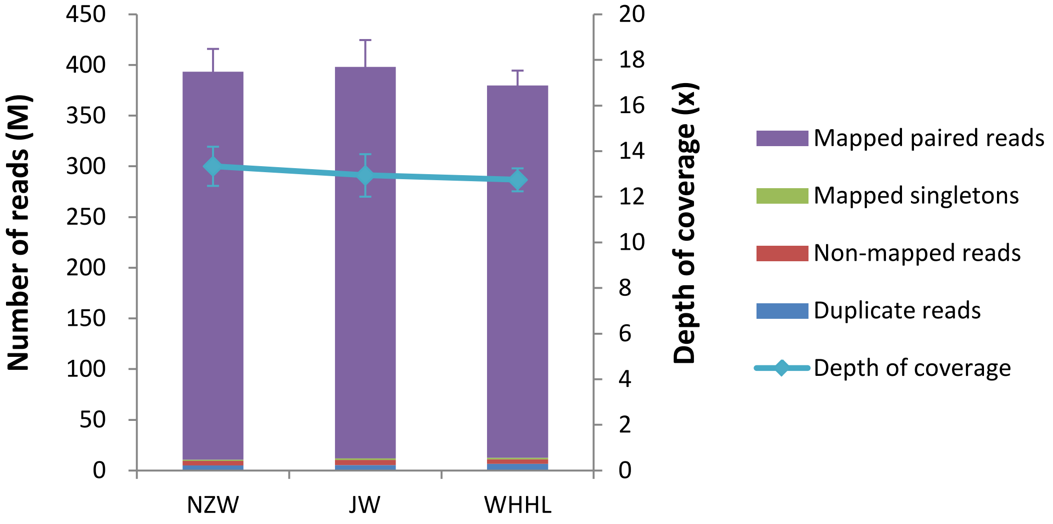

Supplementary Figure S2. Number of reads mapped to genome.

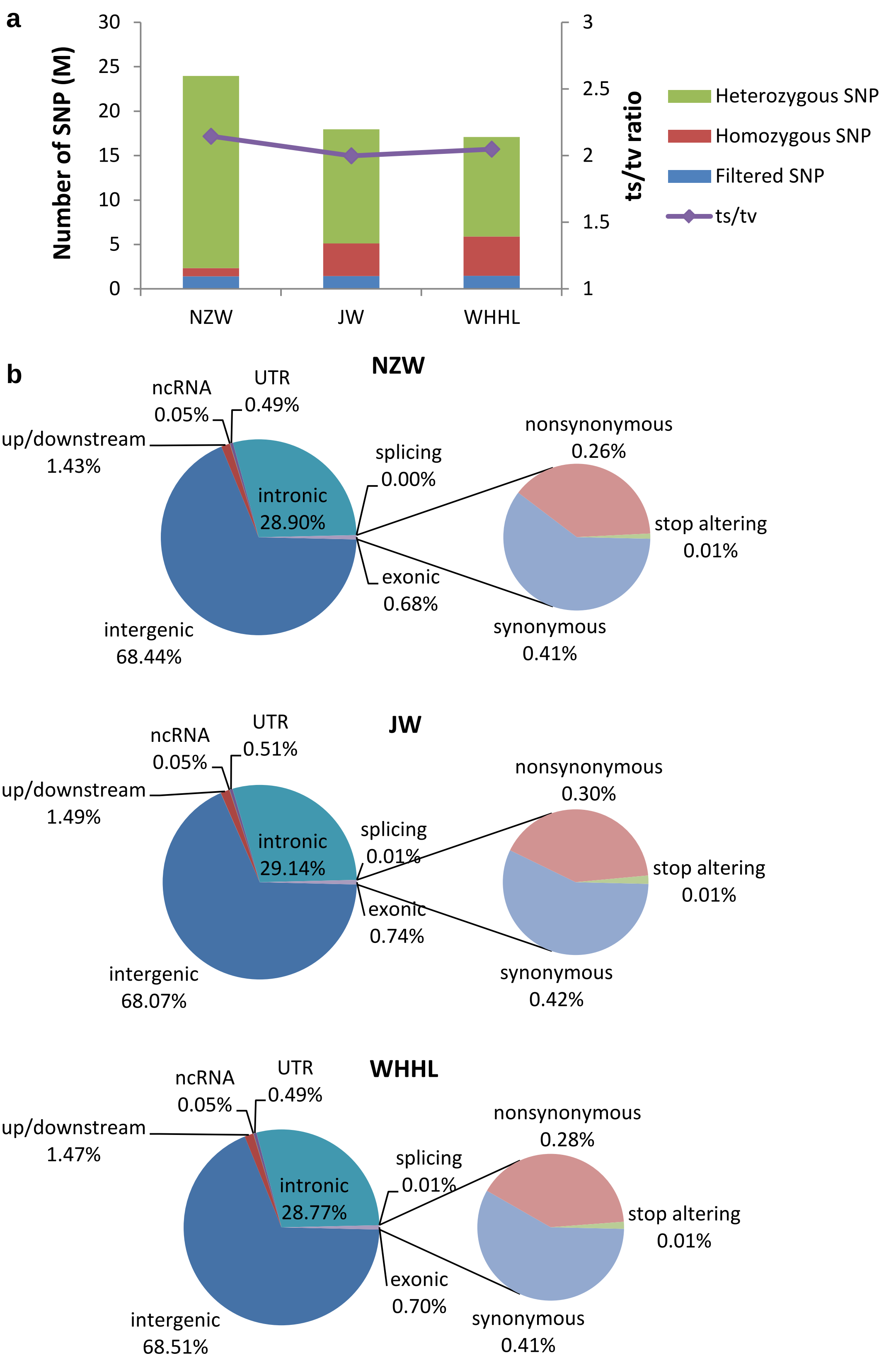

Supplementary Figure S3. Summary of SNP calling and annotation. (a) Number of SNPs called and transition to transversion ratio (ts/tv). (b) Proportion of SNPs in different genomic regions.

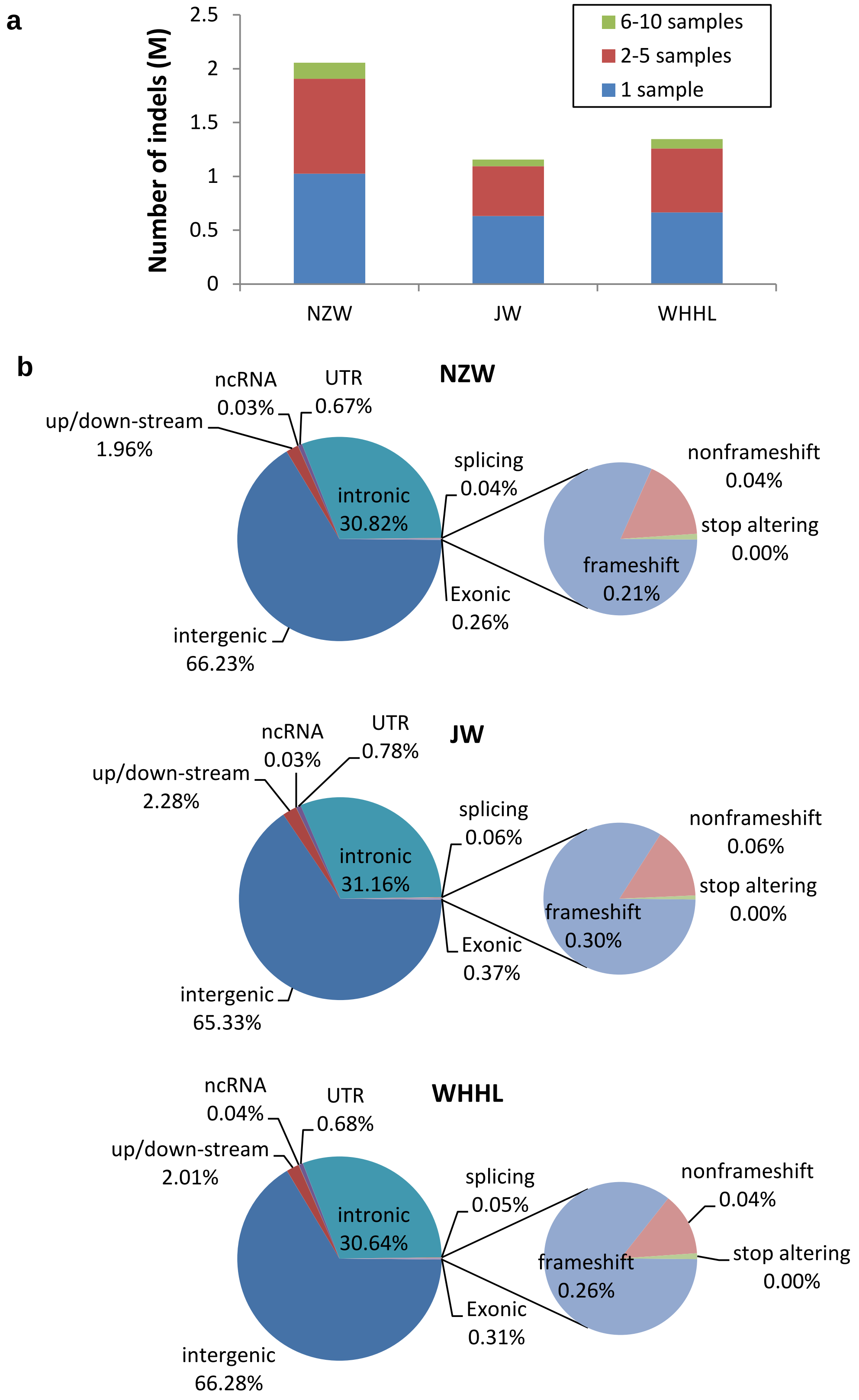

Supplementary Figure S4. Summary of indel calling and annotation. (a) Number of indels called and frequency among samples. (b) Proportion of indels in different genomic regions.

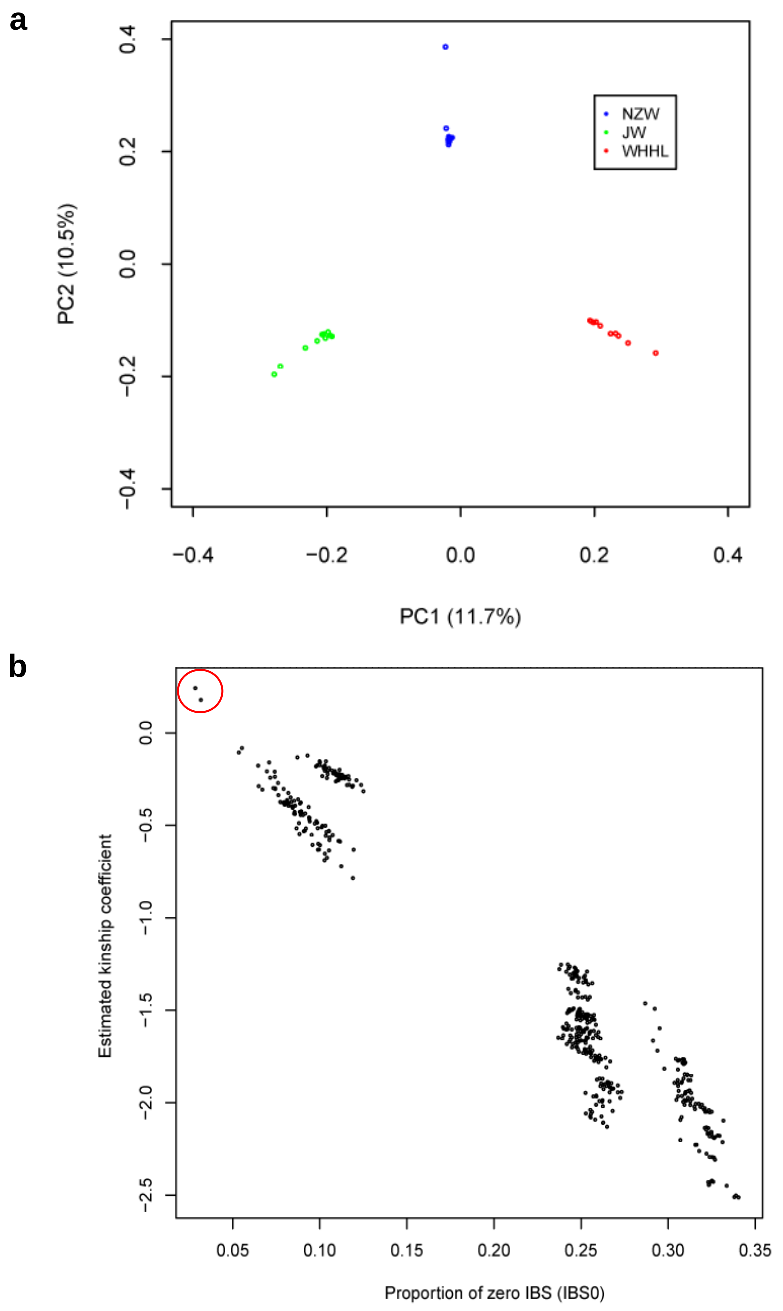

Supplementary Figure S5. Genetic relationship based on whole-genome SNPs. (a) Principal component analysis. The first (PC1) and second component (PC2) are shown in the plot. The percentages indicate the proportion of variance explained by each component. (b) Kinship of the rabbits. Each point represents a rabbit pair. Only two pairs of WHHL rabbits show close genetic relationship.

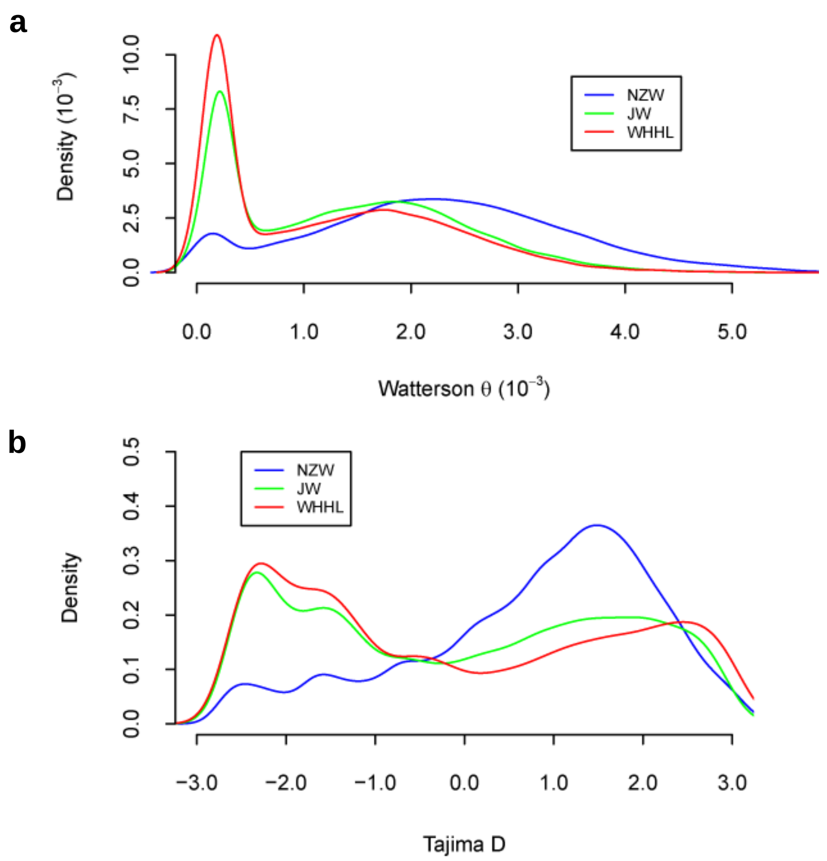

Supplementary Figure S6. Distribution of population diversity statistics. The statistics were calculated for every 100 kb sliding-window across the genome. (a) Watterson's θ. (b) Tajima's D.

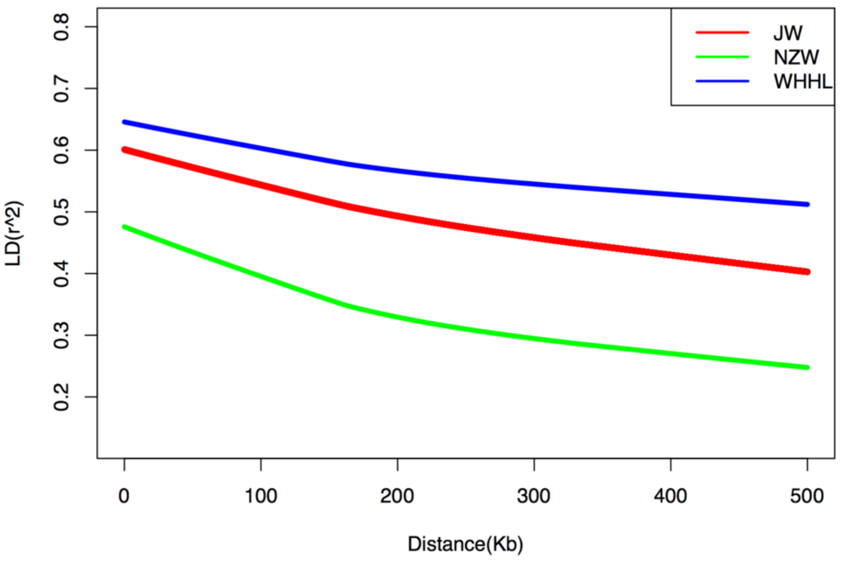

Supplementary Figure S7. Decay of linkage disequilibrium (LD). LD is measured by correlation coefficient (r^2) between two SNP markers.

a

b

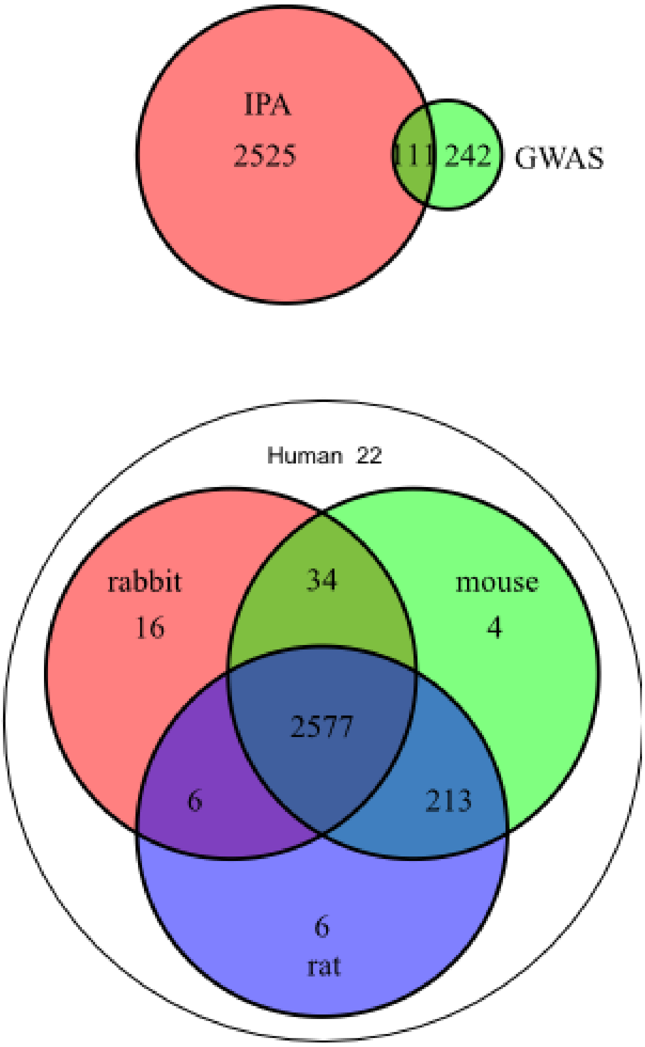

Supplementary Figure S8. Summary of prior genes associated with cardiovascular diseases. (a) The prior genes were collected from the database of Ingenuity Pathway Analysis (IPA) and NHGRI GWAS Catalog. (b) Orthologs of the human prior genes in rabbits, mice and rats retrieved from Ensembl and TreeFam.

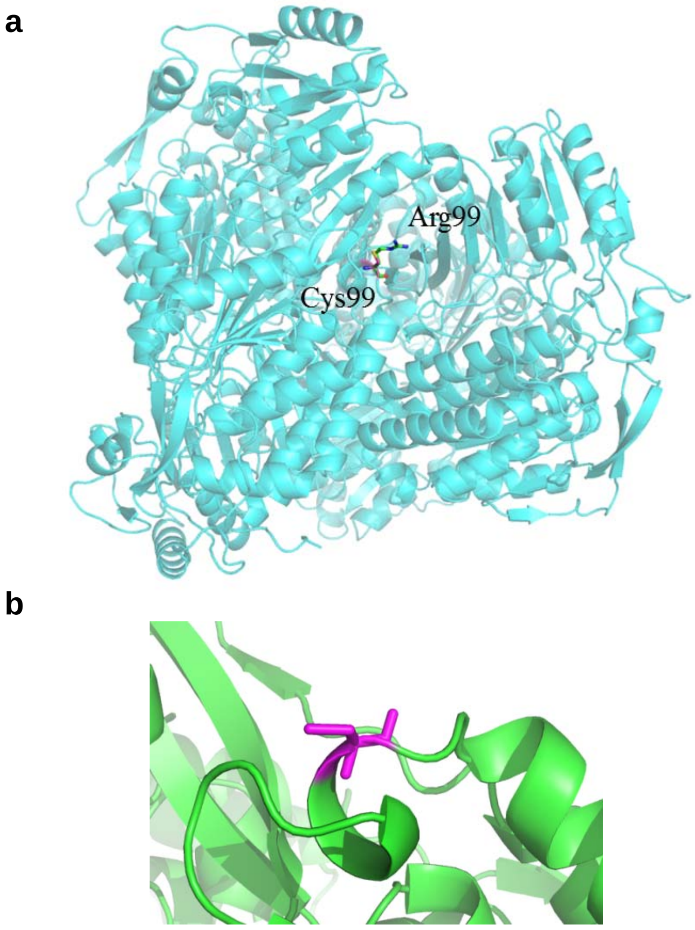

Supplementary Figure S9. Homology modeling structure of ALDH2 for rabbits. (a) The global structure is very similar to that of humans. The mutant residue R99C is represented in stick. (b) Local structure around the mutant residue.

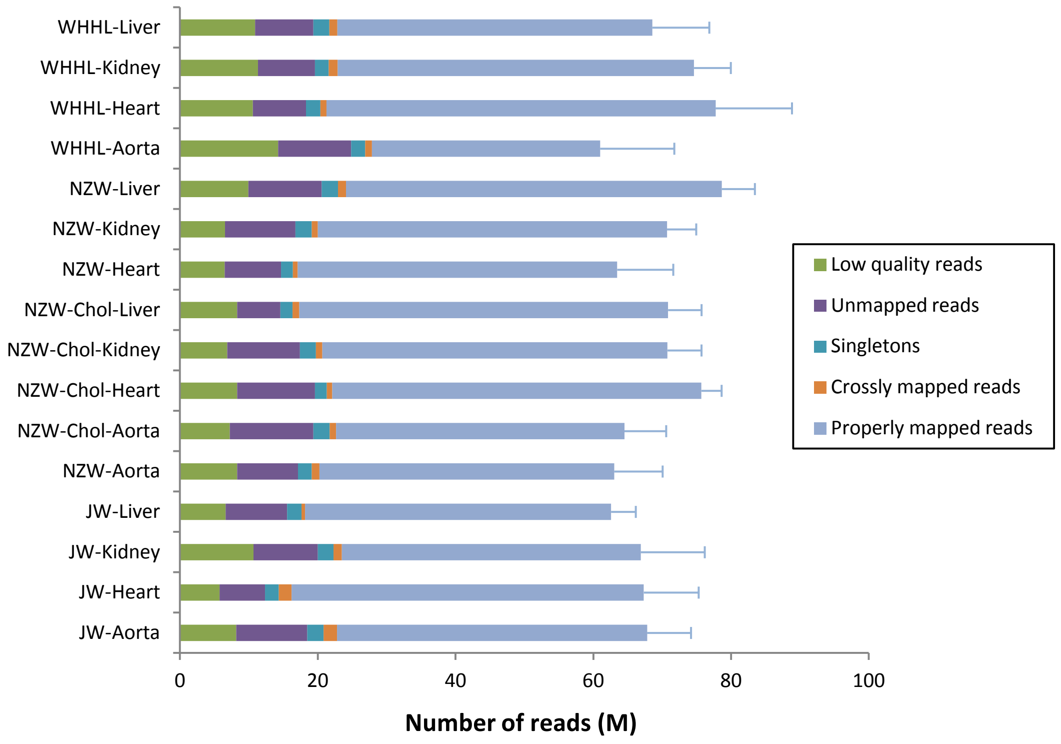

Supplementary Figure S10. Summary of quality control and genome mapping of RNA-Seq data.

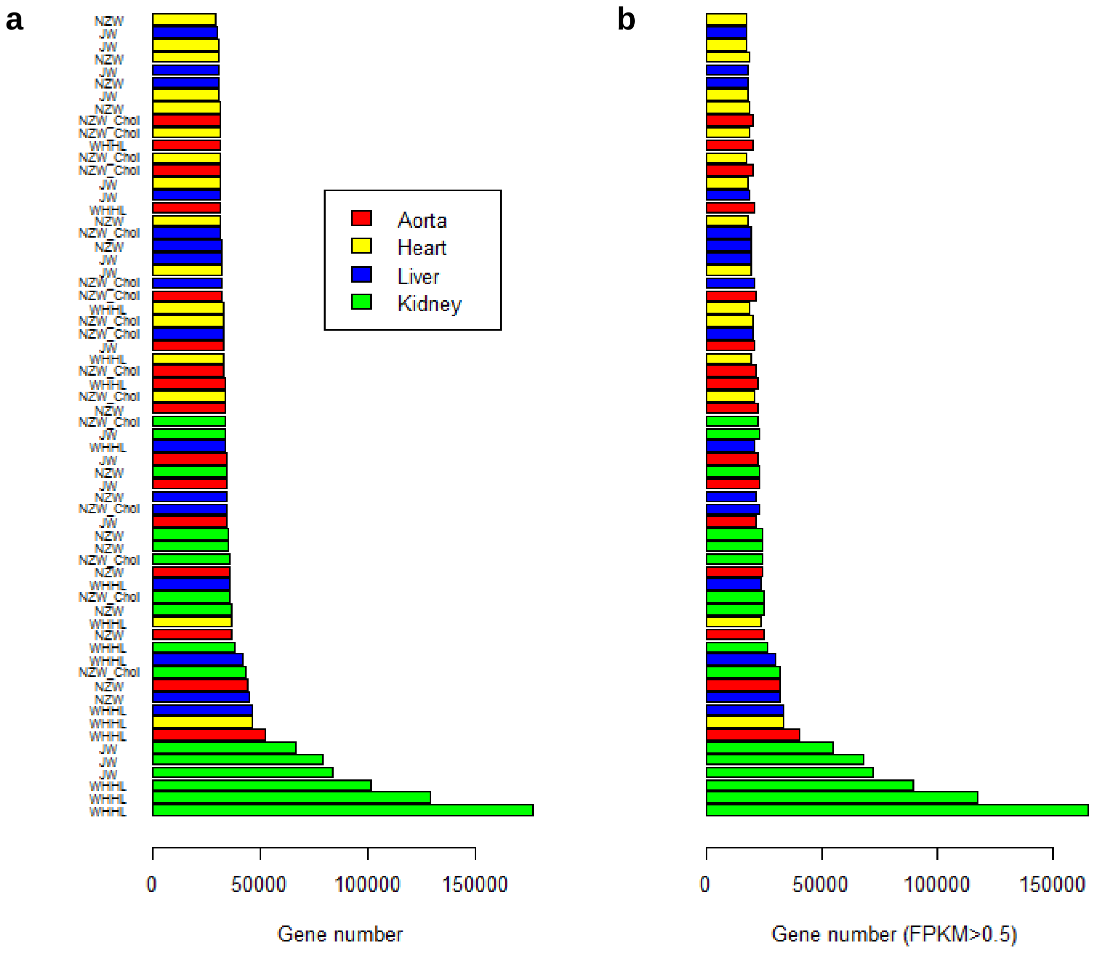

Supplementary Figure S11. Number of genes constructed by Cufflinks for each sample. (a) Total number of genes. Seven samples with gene number greater than 50,000 were not used in Cuffmerge. (b) Number of genes with FPKM > 0.5.

a

b

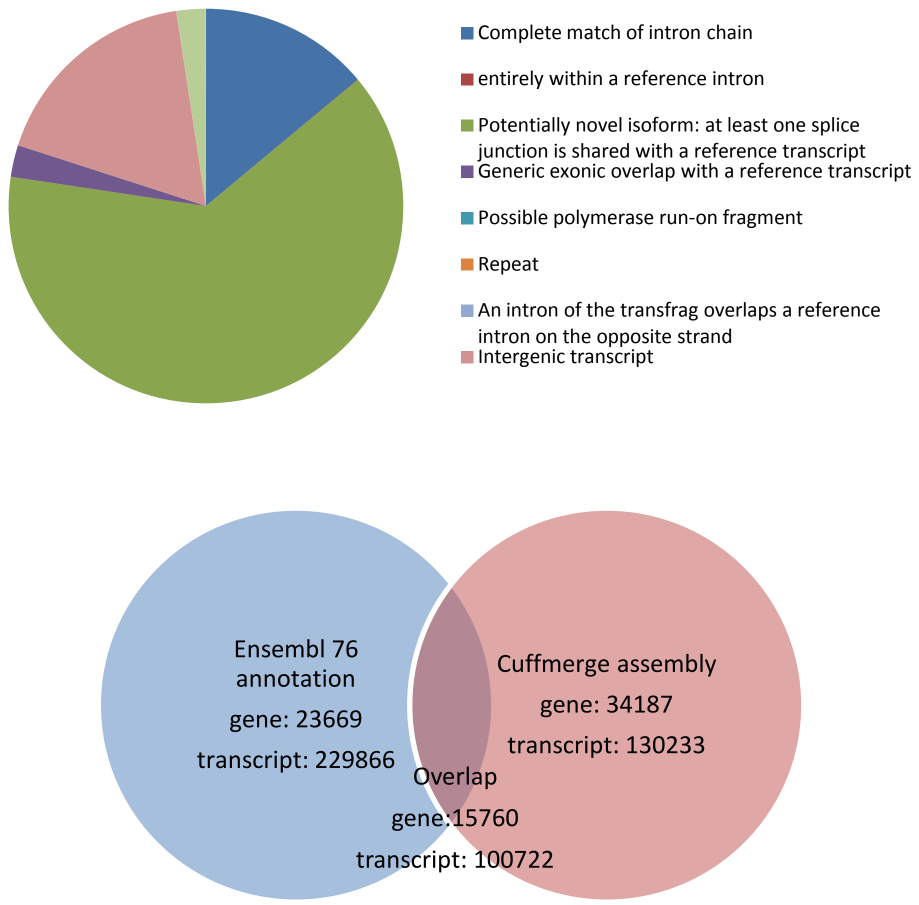

Supplementary Figure S12. Transcriptome merged by Cuffmerge across all samples. (a) Comparison of reconstructed transcripts with the reference (Ensembl 76). (b) Overlap of genes and transcripts between the merged assembly and the reference.

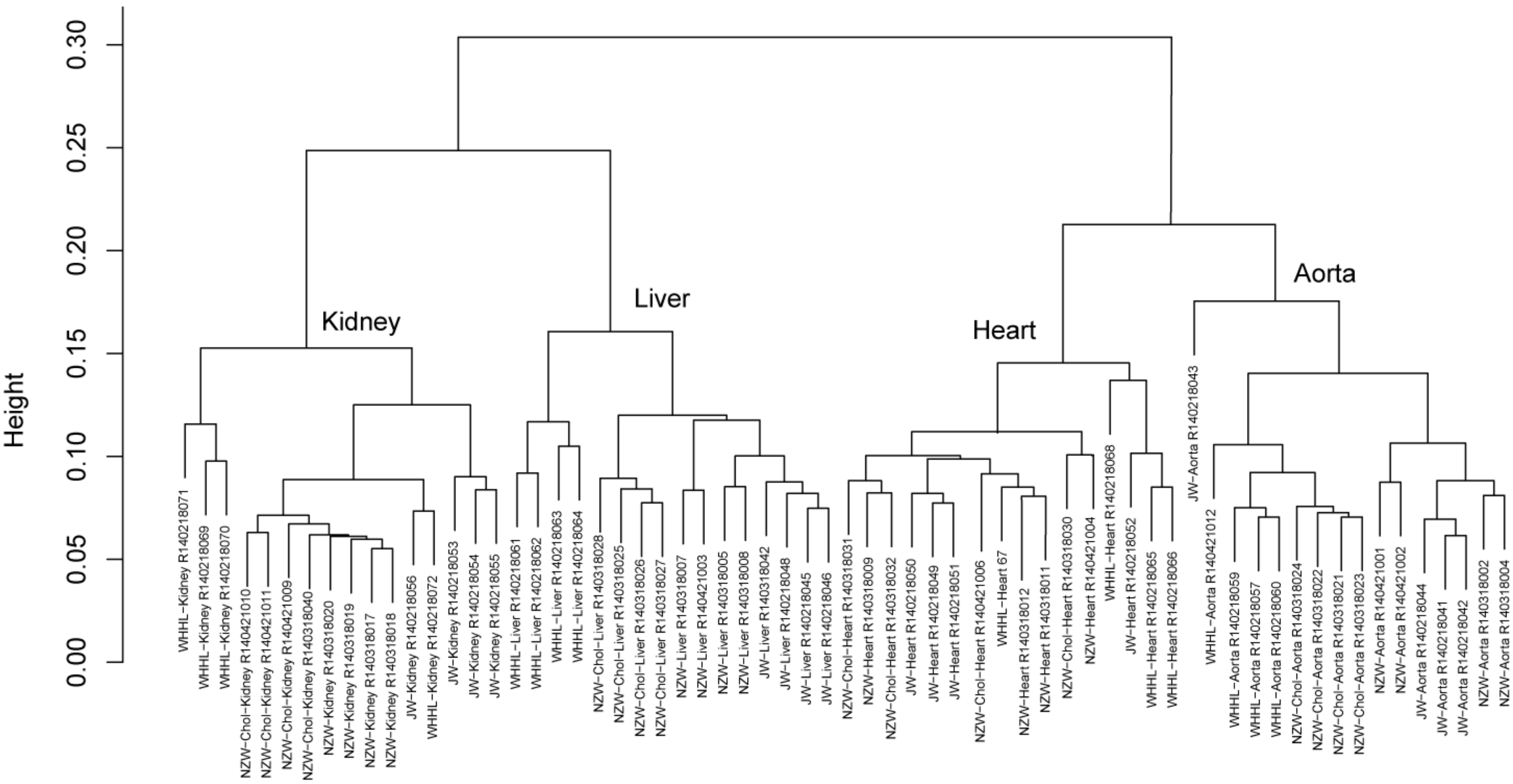

Supplementary Figure S13. Sample clustering based on gene expression profile. The similarity was measured by the correlation coefficient between normalized counts across all genes. Samples from the same tissue are clustered together.

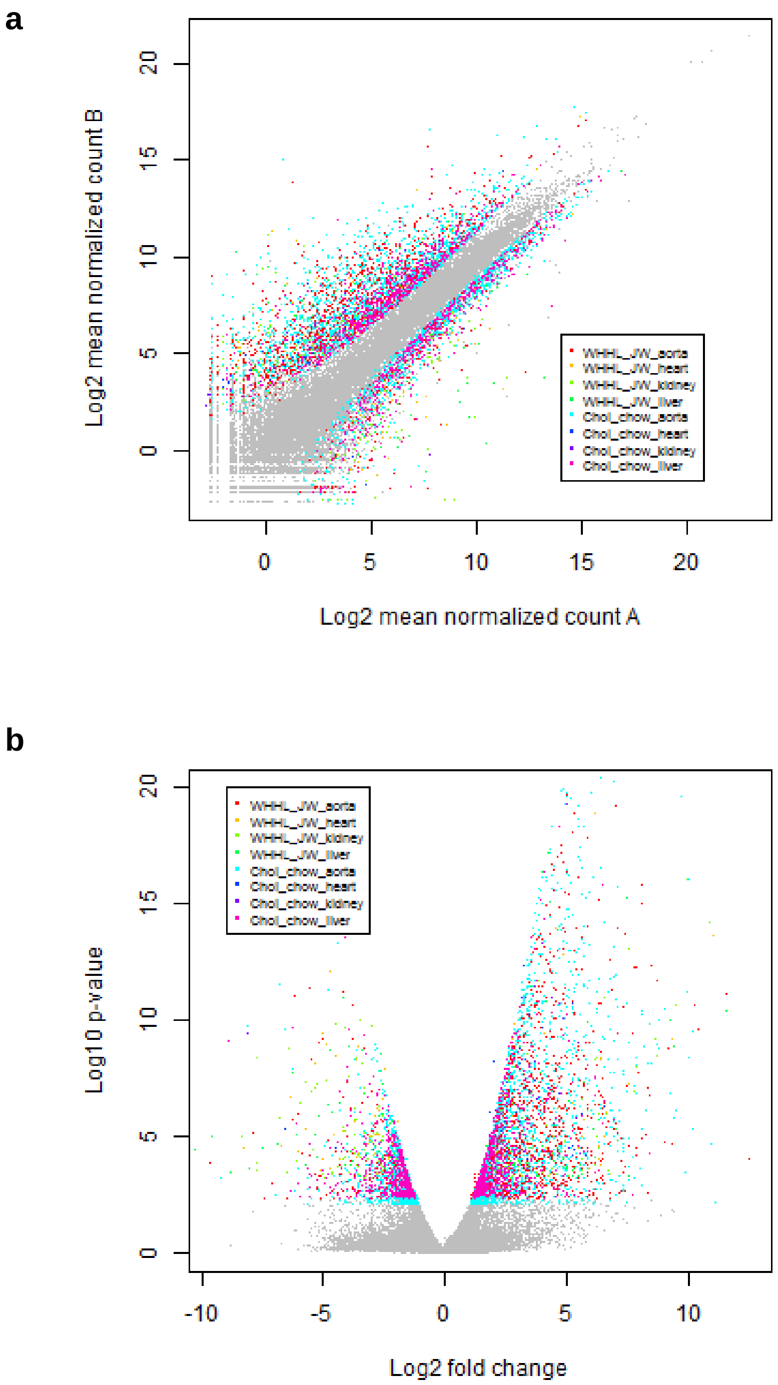

Supplementary Figure S14. Differential expression analysis by DESeq. (a) Pairwise comparison between WHHL and JW, as well as between Chol-fed and chow-fed NZW rabbits for each tissue. (b) Volcano plot for each comparison. Genes with FDR < 0.1 and FDR > 0.1 are represented in colors and grey, respectively.

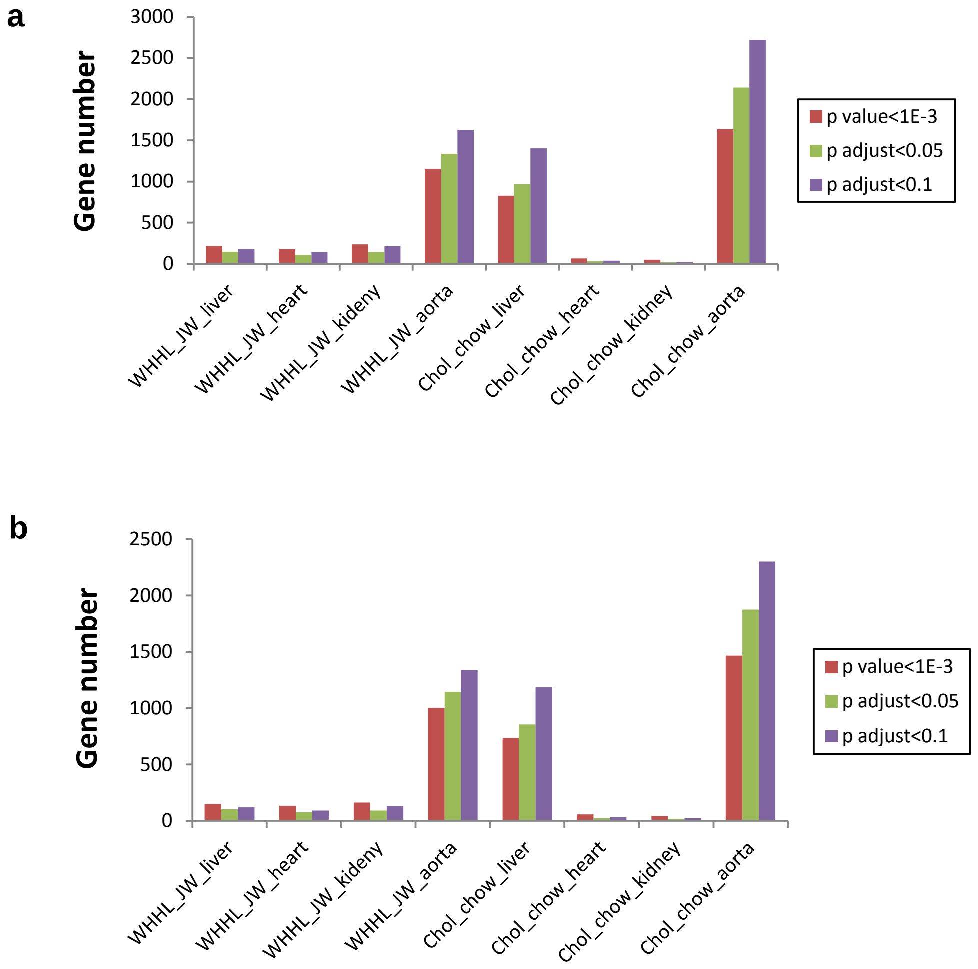

Supplementary Figure S15. Number of differentially expressed genes with different cutoff. (a) Total genes. (b) Genes with known Ensembl ID.

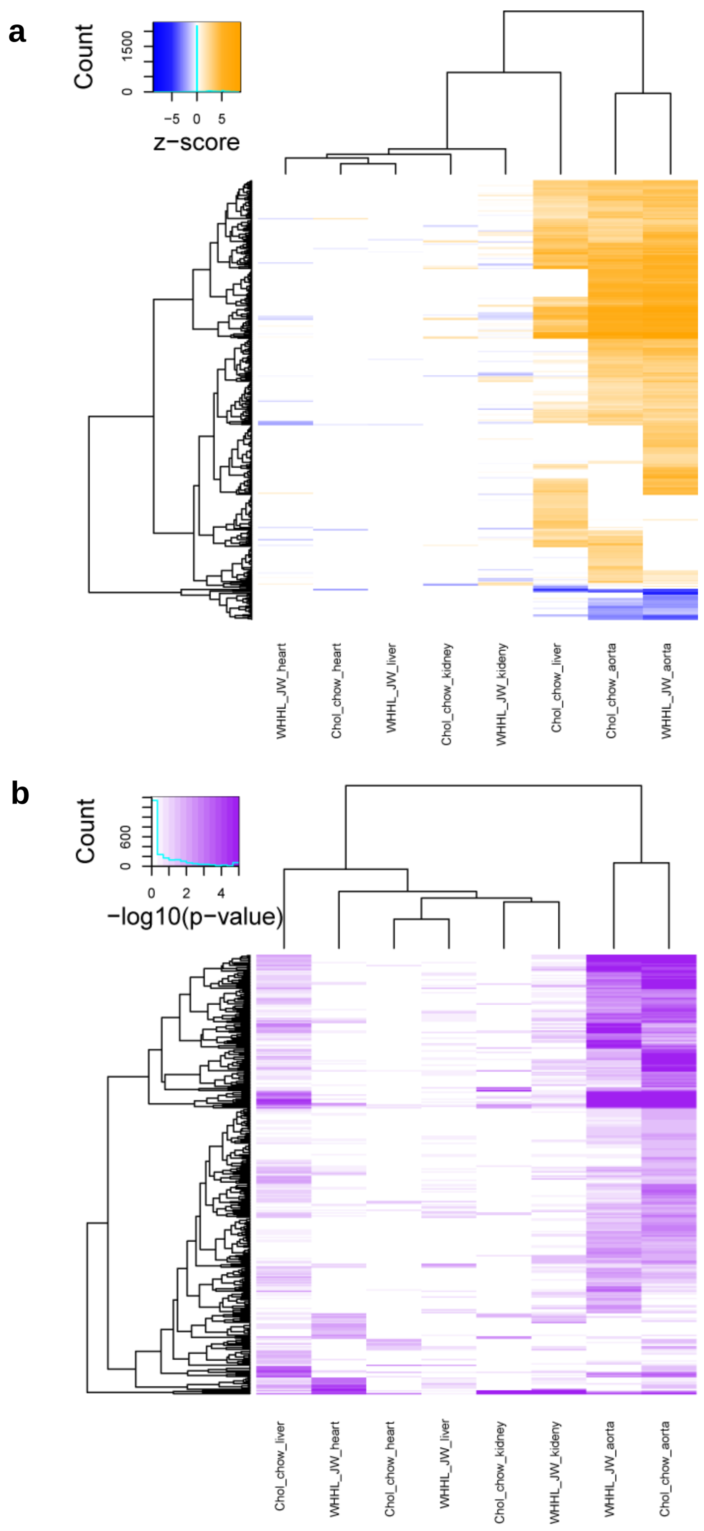

Supplementary Figure S16. Clustering based on results of Ingenuity Pathway Analysis (IPA). (a) Clustering based on significantly activated (z-score > 2) and inhibited (z-score < -2) functional categories in at least one condition. (b) Clustering based on significantly over-represented pathways (P < 0.05) in at least one condition.

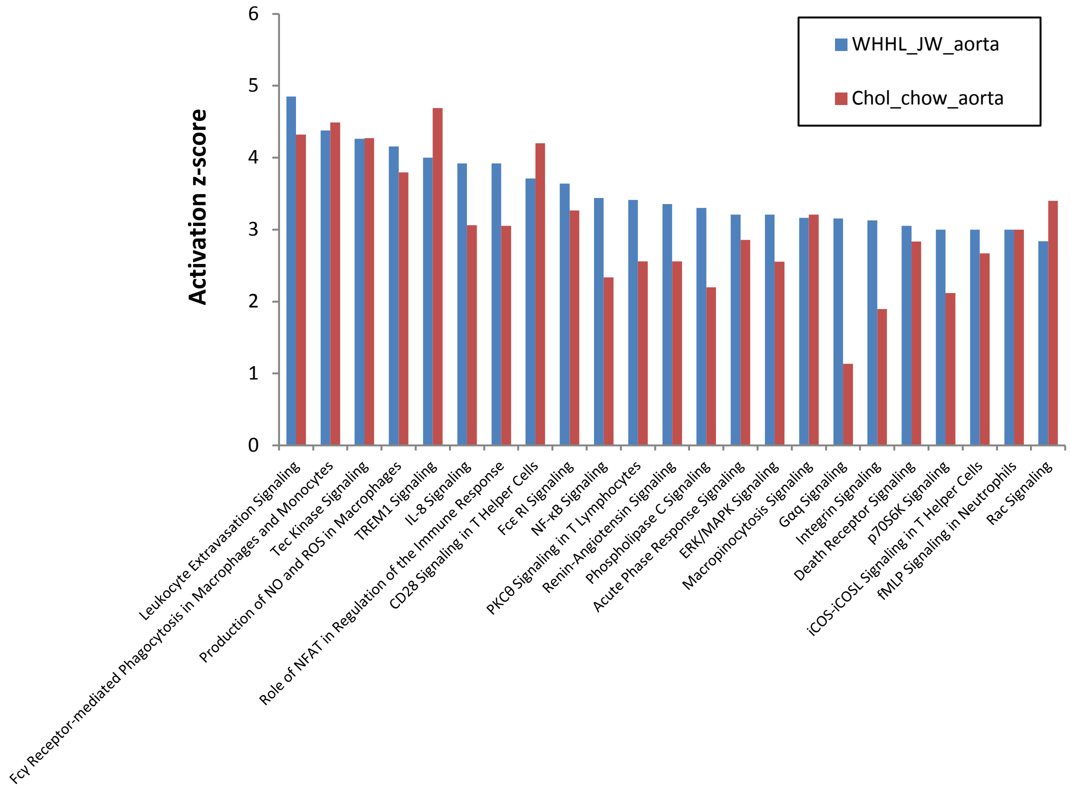

Supplementary Figure S17. Significantly activated pathways in the aorta. Pathways with activation z-score > 3 in WHHL or Chol-fed rabbits are shown.

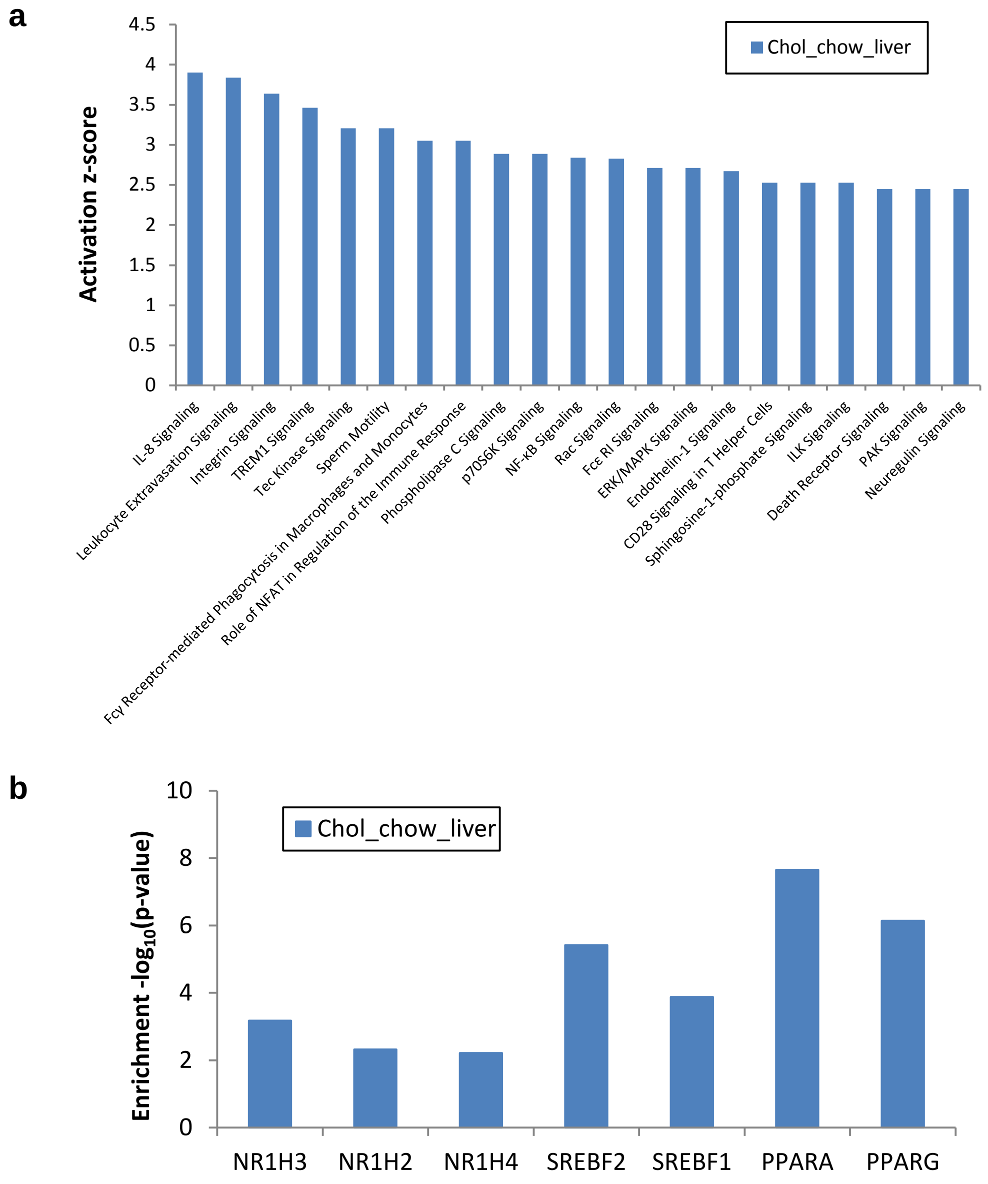

Supplementary Figure S18. Pathway and upstream regulator analysis in the liver. (a) Pathways with activation z-score > 2.5 in Chol-fed rabbits. None of the pathways were observed in WHHL rabbits. (b) Transcriptional regulators for lipid metabolisms indentified by enrichment analysis ($P < 0.05$).

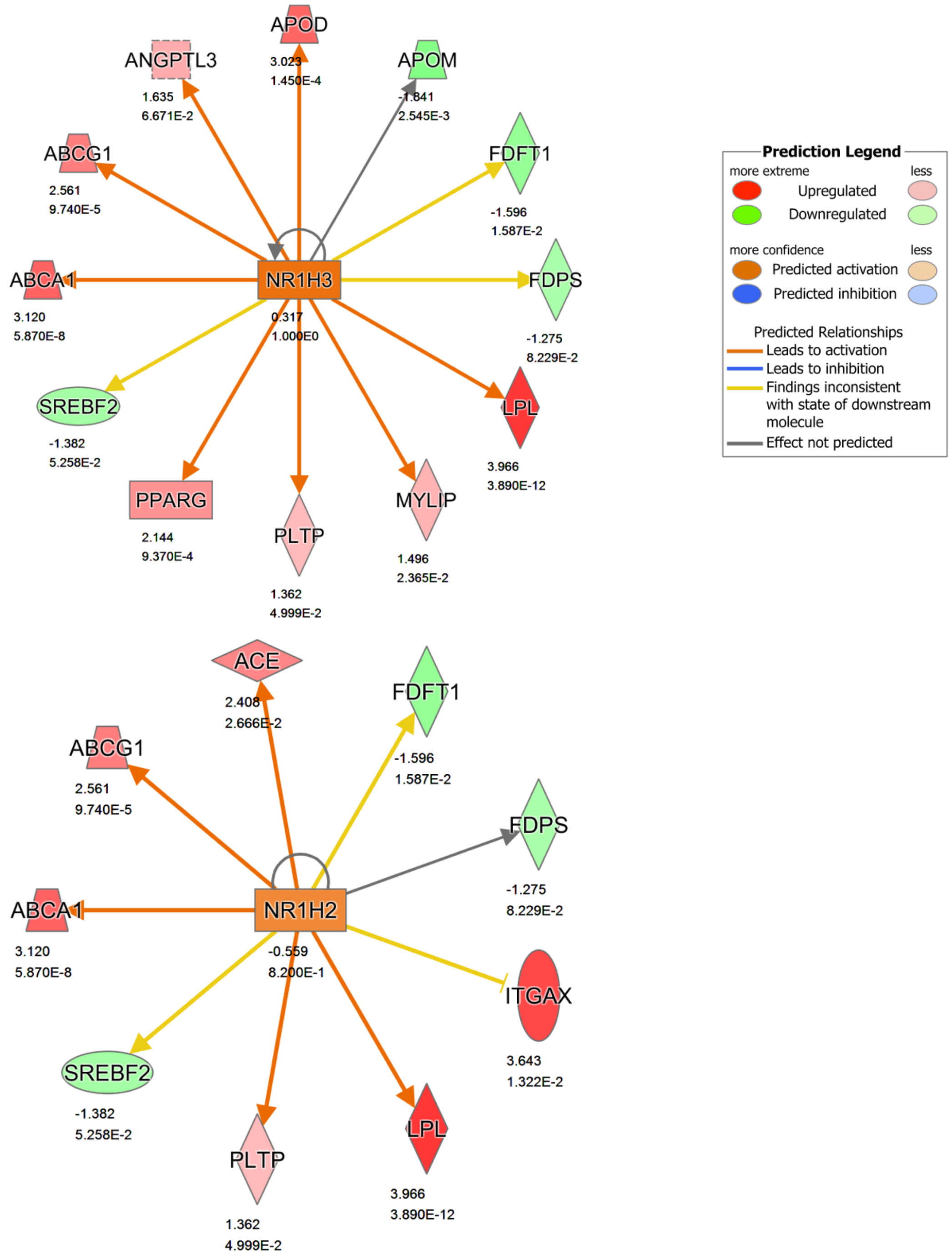

Supplementary Figure S19. Transcriptional regulation by LXRs (NR1H3 and NR1H2) in the liver of Chol-fed rabbits. The numbers beside each gene indicate the $\log_2$-fold change and FDR value, respectively. Differentially expressed target genes (FDR<0.1) are displayed. The networks were generated through the use of IPA.

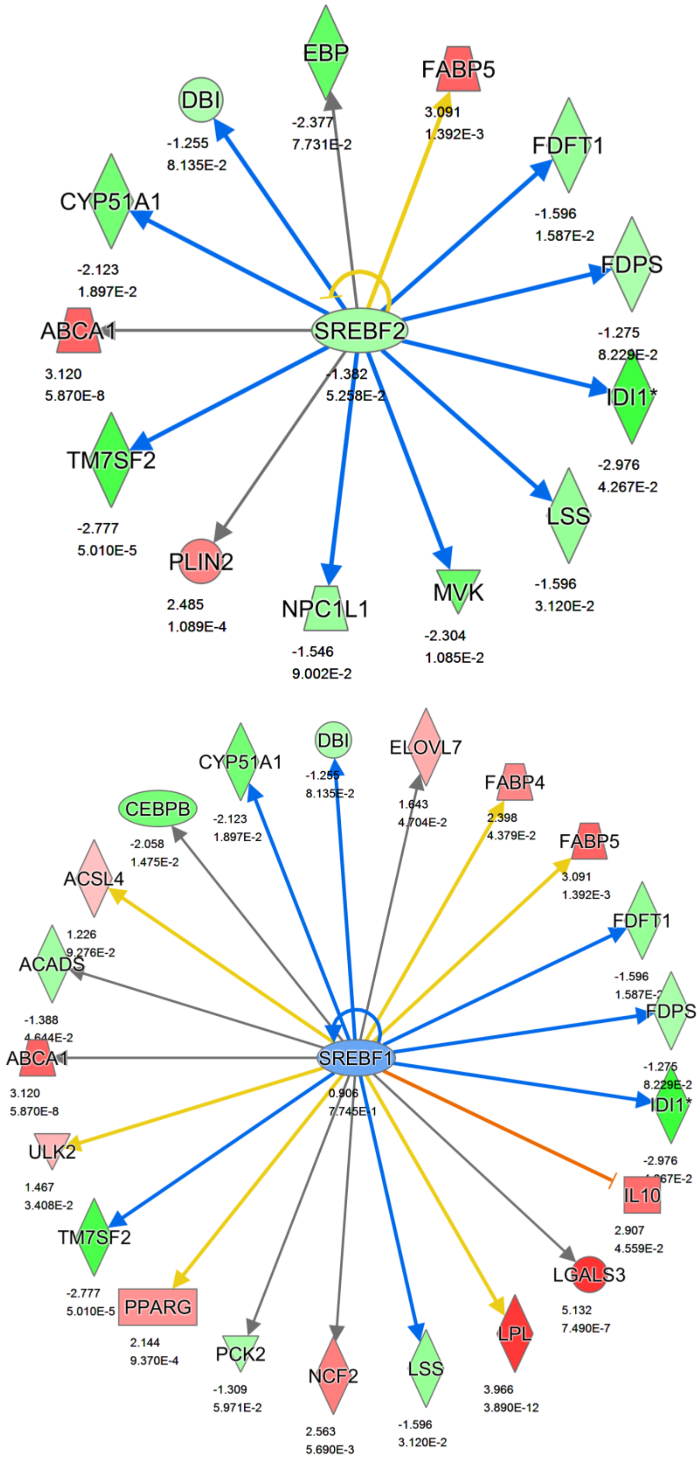

Supplementary Figure S20. Transcriptional regulation by SREBF2 and SREBF1 in the liver of Chol-fed rabbits. Legends are presented in accordance with IPA.

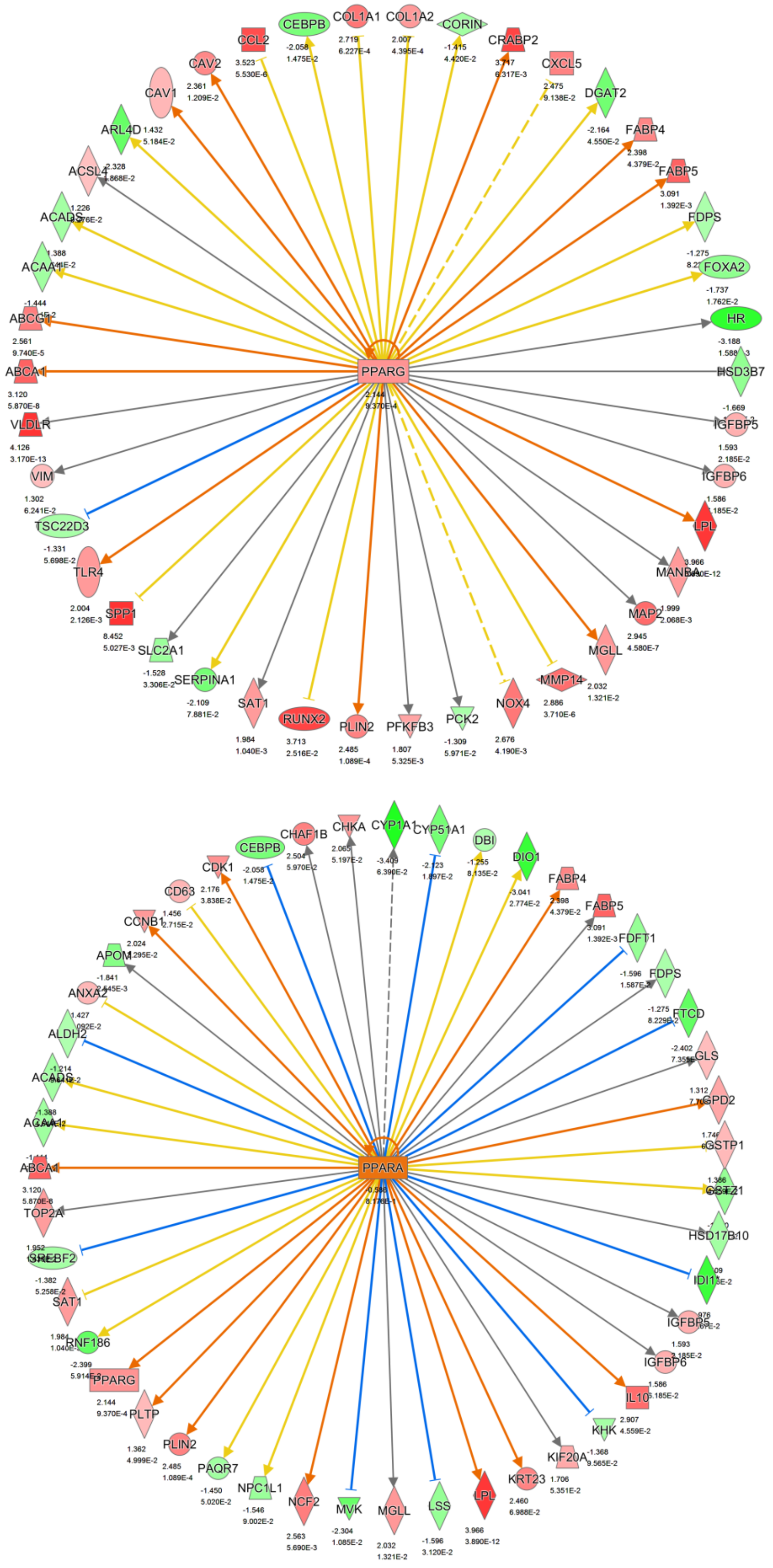

Supplementary Figure S21. Transcriptional regulation by PPARG and PPARA in the liver of Chol-fed rabbits. Legends are presented in accordance with IPA.

Supplementary Table S1. Screening for deleterious mutations with high allele frequency in WHHL rabbits.

Symbol	Chromosome	Position	Reference	Alternative	Type	NZW frequency	JW frequency	WHHL frequency	SIFT score
**LDLR [a]**	chrUn0216	9830	TCCGAGCCGTC(G)	-	(non)frameshift deletion	0	0	1	NA
**RBFOX3**	chrUn0933	5166	-	T	frameshift insertion	0	0	1	NA
**BIRC8**	chr14	5873075	A	C	nonsynonymous SNV	0	0	1	0
**BIRC8**	chr14	5873331	T	G	nonsynonymous SNV	0	0	1	0.027
**NOD1**	chr10	14842319	C	A	nonsynonymous SNV	0	0.05	1	0.013
**ALDH2**	chr21	7756767	C	T	nonsynonymous SNV	0.1	0	1	0.017
**NPY**	chr10	9174216	G	T	nonsynonymous SNV	0.15	0	1	0.049
**RBFOX3**	chrUn0933	5137	GCTGTCACAC	-	frameshift deletion	0.05	0.15	1	NA
**MYRF**	chrUn0019	3028850	G	A	nonsynonymous SNV	0.05	0	0.9	0.04
**SP110**	chr7	172124691	C	T	stopgain SNV	0.05	0.2	1	NA
**VWF**	chr8	32169465	G	T	nonsynonymous SNV	0.25	0	1	0.007
**OLR1**	chr8	28675537	G	A	nonsynonymous SNV	0	0.25	1	0.042
**HTR3D**	chr14	81685343	A	G	nonsynonymous SNV	0	0	0.85	0

**SLC30A3**	chr2	158805362	T	C	nonsynonymous SNV	0	0	0.85	0
**INMT**	chr10	15070273	C	T	nonsynonymous SNV	0	0	0.85	0.01
**CRHR2**	chr10	14986145	C	T	nonsynonymous SNV	0	0	0.85	0.035
**HSPG2**	chr13	131435779	C	T	nonsynonymous SNV	0	0	0.85	0.049
**BCR**	chrUn0112	215181	-	CGGCGGCGTCGGCGGCGT	nonframeshift insertion	0.3	0	1	NA
**CD44**	chr1	174512129	A	C	nonsynonymous SNV	0.2	0	0.9	0.028
**ZNF274**	chrUn0065	1247531	A	G	nonsynonymous SNV	0.1	0	0.85	0
**SERPINF1**	chr19	16393505	G	A	nonsynonymous SNV	0	0	0.8	0.016
**RBFOX3**	chrUn0933	5160	T	C	nonsynonymous SNV	0.2	0.2	1	0.016
**GPD1L**	chr14	19278955	C	T	stopgain SNV	0.15	0.05	0.85	NA
**GRIP1**	chr4	45458751	C	G	nonsynonymous SNV	0	0	0.75	0.016
**DOCK4**	chr7	48929100	C	T	nonsynonymous SNV	0	0	0.7	0.013

[a] WHHL rabbits should have a 12-bp in-frame deletion in LDLR. However, the current reference assembly misses one base here, resulting in a 11-bp deletion called.
